# Defining the Blood Cytokine Profile in Asthma to Understand Asthma Heterogeneity

**DOI:** 10.1002/iid3.70116

**Published:** 2025-03-19

**Authors:** Karina Bingham, Yousef Al Zahrani, Iain Stewart, Michael A. Portelli, Andrew Fogarty, Tricia M. McKeever, Ananga Singapuri, Liam G. Heaney, Adel H. Mansur, Rekha Chaudhuri, Neil C. Thomson, John W. Holloway, Peter H. Howarth, Ratko Djukanovic, John D. Blakey, Anoop Chauhan, Christopher E. Brightling, Zara E. K. Pogson, Ian P. Hall, Luisa Martinez‐Pomares, Dominick Shaw, Ian Sayers

**Affiliations:** ^1^ Centre for Respiratory Research, NIHR Nottingham Biomedical Research Centre, School of Medicine, Biodiscovery Institute University of Nottingham Nottingham UK; ^2^ Respiratory Care Department Prince Sultan Military College of Health Sciences Dhahran Saudi Arabia; ^3^ Faculty of Medicine National Heart & Lung Institute Imperial College London UK; ^4^ Division of Epidemiology and Public Health University of Nottingham Nottingham UK; ^5^ Institute for Lung Health University of Leicester, Glenfield Hospital Leicester UK; ^6^ Centre for Infection and Immunity Queen's University of Belfast Belfast UK; ^7^ Respiratory Medicine Birmingham Heartlands Hospital Birmingham UK; ^8^ Institute of Infection, Immunity and Inflammation University of Glasgow Glasgow UK; ^9^ NIHR Southampton Biomedical Research Centre University Hospital Southampton Southampton UK; ^10^ Clinical & Experimental Sciences, Faculty of Medicine University of Southampton Southampton UK; ^11^ Medical School Curtin University Perth Western Australia; ^12^ Respiratory Medicine Sir Charles Gairdner Hospital Perth Western Australia Australia; ^13^ Research and Innovation, Portsmouth Hospitals University NHS Trust Portsmouth UK; ^14^ Department of Health Sciences University of Leicester Leicester UK; ^15^ United Lincolnshire Hospitals NHS Trust Lincoln County Hospital Lincoln UK; ^16^ School of Life Sciences, Faculty of Medicine and Health Sciences University of Nottingham Nottingham UK

**Keywords:** asthma, blood cytokines, endotypes, genetics

## Abstract

**Background:**

Asthma is a heterogeneous disease characterized by overlapping clinical and inflammatory features.

**Objective:**

This study aimed to provide insight into the systemic inflammatory profile in asthma, greater understanding of asthma endotypes and the contribution of genetic risk factors to both.

**Methods:**

4205 patients with asthma aged 16–60 were recruited from UK centers; serum cytokines were quantified from 708, including cytokines associated with Type 1, 2 and 17 inflammation. 3037 patients were genotyped for 25 single nucleotide polymorphisms associated with moderate‐severe asthma.

**Results:**

Serum cytokines associated with Th2 inflammation showed high coordinated expression for example, IL‐4/IL‐5 (*R* ^2^ = 0.513). The upper quartile of the serum cytokine data identified 43.7% of patients had high levels for multiple Th2 cytokines. However, the groups defined by serum cytokine profile were not clinically different. Childhood‐onset asthma was characterized by elevated total IgE, allergic rhinitis and dermatitis. Exacerbation prone patients had a higher BMI, smoking pack‐years, asthma control questionnaire score and reduced lung function. Patients with blood eosinophils of > 300 cells/µL had elevated total IgE and lower smoking pack‐years. None of these groups had a differential serum cytokine profile. Asthma risk alleles for; rs61816764 (*FLG*) and rs9303277 (*IKFZ3*) were associated with childhood onset disease (*p* = 2.67 × 10^−^^4^ and 2.20 × 10^−^^7^; retrospectively). No genetic variant was associated with cytokine levels.

**Conclusion:**

Systemic inflammation in asthma is complex. Patients had multiple overlapping inflammatory profiles suggesting several disease mechanisms. Genetic risk factors for moderate‐severe asthma confirmed previous associations with childhood onset of asthma.

## Summary

- A large proportion of asthma patients showed a complex systemic inflammation with multiple overlapping inflammatory profiles suggesting several disease mechanisms spanning Type 1, 2 and 17 inflammation.
- Nearly half of asthma patients showed elevated levels of mediators that are current biological drug targets suggesting these patients may be amenable to these drugs or even combination monoclonal antibody therapy.
- Our data also confirms and extends previous findings on childhood versus adult onset, exacerbation prone asthma and eosinophilic driven asthma subgroups.

## Graphical abstract

Asthma patients showed a heterogeneous systemic inflammation profile with overlapping inflammatory profile. Additionally, The cytokines demonstrated high coordinated expression."

**Abbreviations**
ACQ: asthma control questionnaire
FEV_1_
: forced expiratory volume in 1 s
FVC: forced vital capacity
GASP: genetics of asthma severity and phenotypes
GINA: global initiative for asthma
GWAS: genome wide association study
ICS: inhaled corticosteroids
OCS: oral corticosteroids
PEF: peak expiratory flow
SNP: single‐nucleotide polymorphism

## 1 Introduction

Asthma is a heterogeneous disease with overlapping immunological, clinical and genetic features. Asthma endotypes describe distinct pathophysiological mechanism at the cellular and molecular level [1, 2]. Previously reported subgroups included Type‐2‐high and Type‐2‐low asthma. Type‐2‐high asthma has been characterized by elevated airway or blood eosinophils and high levels of Th2 cytokines IL‐4, IL‐5 and IL‐13 in the airways produced by both T cells and ILCs. Type 2 immune responses in asthma are initiated by master regulatory cytokines called alarmins which include IL‐33 and thymic stromal lymphopoietin (TSLP). These alarmins are released by epithelial cells in response to an environmental stimulus that is, virus. Th2 cytokines IL‐4, IL‐5 and IL‐13 drive allergic response by promoting IgE production, eosinophil activation and tissue remodeling [3, 4, 5, 6]. Type‐2‐low asthma is less well defined and may have Type 1 (Th1) (IFN‐ƴ and IL‐6) and/or Type 17 (Th17) (IL‐17A) inflammation. IFN‐ƴ is crucial for promoting cell‐mediated immunity, activating macrophages and sustaining Th1‐driven inflammation. IL‐6 plays a central role in immune responses, acute inflammation and chronic inflammation. Whereas IL‐17 is believed to recruit neutrophils and monocytes and play a role in acute and chronic inflammation [7, 8]. A recent moderate‐severe asthma genome wide association study (GWAS) discovered 25 genome wide significant signals. Many of these genes are in loci where the signals suggest genes/pathways that are involved in the innate and adaptive immune responses, for example, IL‐33, IL‐4/5/13, STAT6, GATA3 and TSLP [9].

Within the last decade newly developed asthma monoclonal antibody therapies targeting specific components of the Type‐2 inflammatory pathway have been brought to clinic for example, anti‐IL‐5 (Mepolizumab), anti‐IL‐4/13 (Dupilumab) demonstrating steroid sparing effects and efficacy in reducing exacerbations [10, 11, 12]. Identifying which patients have different types of inflammation in a noninvasive way that is also amenable to targeting by specific biologics is important as it could improve personalized therapeutic approaches.

However, a greater understanding of both airway and systemic inflammation in asthma is needed to further define drivers of the disease. Particularly in defining asthma subgroups including childhood onset and exacerbation prone asthma patients especially as some patients are still prone to exacerbations despite taking inhaled corticosteroid (ICS) treatment [10, 12, 13]. Although clinical studies have shown promising results with monoclonal antibodies, many patients still do not respond to these drugs [14, 15].

Previous work looking at biomarkers in the upper and lower airways in asthma patients has shown some promising biomarker candidates and correlation matrices that may reflect underlying asthma endotypes, for example, sputum eosinophils and/or fractional exhaled nitric oxide, reviewed [16]. Similarly, different‐omic approaches has been used to identify different patient clusters particularly in cohorts such as uBioPRED using several sample types but particularly sputum [17]. Yet other asthma sputum studies have struggled with identifying endotypes due to the heterogeneity in airways amongst asthma patients [18]. Measuring cytokines in the serum of asthma patients is not commonly used however, cytokines are key to recently developed asthma monoclonal antibody therapies. Some of these therapies may not be effective in all asthma patients, therefore measuring cytokine levels could provide useful information for understanding the pathologic process and monitoring of disease progression and inflammation.

We therefore hypothesized that measuring cytokines in sera associated with Th1, Th2 and Th17 inflammation could (a) provide insight into asthma inflammatory profiling, (b) provide greater understanding of recognized asthma subgroups including childhood versus early/older adult‐onset disease, exacerbation prone and eosinophilic asthma, and (c) be driven by genetic risk factors for moderate‐severe asthma or drive these recognized asthma subgroups.

## 2 Methods

See Online Supporting Information S1: Supplement for additional methods.

### 2.1 Subjects

Patients aged 16–60 were recruited from across 18 UK centers with physician‐diagnosed asthma of a minimum of 1 year and on current prescription for asthma (see methods section in the supplement for further detail on the inclusion and exclusion criteria and information on the Genetics of Asthma and Severe Phenotypes [GASP] cohort). 708 asthma patients were used for the serum biomarker analysis, 4,205 patients were used for the clinical analysis with 3037 used for the clinical and genetic analysis (Table 1).

**Table 1:** Demographics, clinical and immunological features of the three patient cohorts used for analyses. Data is presented as mean and standard deviation (SD), median and minimum and maximum (Min‐Max) or Number (*n*) and percentage (%).

Cohort	Clinical	Genetics	Serum
Subjects (*n*)	4205	3037	708
Age seen (years: Mean and SD)	42.0 (11.93)	43.10 (11.45)	41.0 (11.84)
	*n* = 3534	*n* = 2597	*n* = 695
Age of onset (years: Mean and SD)*	18.60 (15.66)	18.70 (15.61)	17.60 (15.92)
	*n* = 2137	*n* = 1766	*n* = 639
Age of onset (*n*):*	2217	1,766	639
< 16	1090 (51.0%)	896 (50.7%)	341 (53.4%)
16–34	619 (29%)	513 (29%)	176 (27.5%)
> 35	428 (20%)	357 (11.8%)	122 (19.1%)
Sex (*n*):	3,875	2849	702
Female	2553 (65.9%)	1922 (67.5%)	500 (71.2%)
BMI (Kg/m^2^: Median & Min‐Max)	27.89 (14.84–78.16)	28.45 (14.84–78.16)	29.35 (14.84–78.16)
	*n* = 2739	*n* = 2048	*n* = 595
Smoking status (*n*):	3279	2438	699
Never	2116 (64.5%)	1576 (64.6%)	528 (75.5%)
Ex‐smoker	737 (22.5%)	543 (22.3%)	159 (22.7%)
Current	426 (13.0%)	319 (13.1%)	12 (1.7%)
Smoking pack years (median and Min‐Max)	6 (0.3–161)	7 (0.03–161)	4 (0.05–10)
	*n* = 936	*n* = 645	*n* = 156
FEV_1_ (%Pred) (Mean and SD)	82.03 (22.21)	80.49 (22.33)	82.07 (21.14)
	*n* = 3000	n = 2,220	n = 669
FEV_1_FVC (Mean and SD)	0.72 (0.13)	0.71 (0.13)	0.73 (0.12)
	*n* = 2682	*n* = 2203	*n* = 558
Blood total IgE (KU/L: Median and Min‐Max)	130.0 (0.60–4900)	118.0 (0.60–4900)	120.0 (2–4850)
	*n* = 1879	*n* = 1325	*n* = 336
Blood Eosinophils (×10^9^/L: Median and Min‐Max)	0.21 (0.0–5.42)	0.21 (0.0–5.42)	—
	*n* = 811	*n* = 731	
Allergic rhinitis (*n*):**	2193	1520	531
Yes	1331 (60.7%)	926 (60.9%)	372 (70.1%)
Atopic dermatitis (*n*):**	1206	756	—
Yes	453 (37.6%)	259 (34.3%)	
Hospital admissions (*n*):	1114	904	641
Never	762 (68.4%)	603 (66.7%)	407 (63.5%)
Once	185 (16.6%)	164 (18.1%)	135 (21.1%)
More than once	104 (9.3%)	85 (9.4%)	65 (10.1%)
High dependency/ICU	63 (5.7%)	52 (5.8%)	34 (5.3%)
Mean ACQ Score 7 (Mean and SD)	1.94 (1.27)	1.96 (1.25)	2.25 (1.21)
	*n* = 1016	*n* = 837	*n* = 587
Mean ACQ Score 6 (Mean and SD)	1.94 (1.36)	1.97 (1.34)	2.27 (1.30)
	*n* = 1052	*n* = 863	*n* = 609
GINA (*n*):	1772	1415	674
1 (Mild)	114 (6.4%)	90 (6.4%)	13 (1.9%)
2 (Mild/moderate)	269 (15.2%)	196 (13.9%)	63 (9.3%)
3/4 (Moderate/severe)	1176 (66.4%)	956 (67.6%)	474 (70.3%)
5 (Severe)	213 (12%)	173 (12.2%)	124 (18.4%)

Abbreviations: ACQ, asthma control questionnaire; FEV1/FVC, forced expiratory volume in 1 s/forced vital capacity ratio; FEV1pp, forced expiratory volume in 1 s percentage predicted; GINA, global initiative for asthma; IgE, immunoglobulin E.

* Age of onset‐some only known as childhood onset not the actual age so marked as < 16 therefore numbers vary for both variables.

** Allergic rhinitis and atopic dermatitis are doctor diagnosis and/or self‐reported.

### 2.2 Collection of Clinical and Immunological Patient Data

Demographics including age, sex, age of onset, height, and weight, along with clinical data baseline lung function (FEV_1_, FVC and PEF) after 4 h withholding reliever medication, smoking status, smoking pack years, current asthma medication, Global Initiative for Asthma (GINA) classification 2018 [19] and ACQ score (see supplement methods section for further details) were recorded. Total serum IgE, number of hospital admissions within the last year, comorbidities such as allergic rhinitis and allergic dermatitis and blood eosinophil count were also recorded.

### 2.3 Collection and Processing of Serum Samples

708 asthma patients, representative of the larger cohort (Table 1) had blood for serum collected when their asthma was stable. Serum separating tubes were centrifuged at 3000 rpm for 10 min at recruitment site and aliquots were made and stored at −80°C. Aliquots of serum samples were sent on dry ice and stored at −80°C at the University of Nottingham Respiratory Medicine laboratories.

### 2.4 Luminex Assay

R&D systems Magnetic Luminex was used to measure the concentration of Th1 (IFN‐y), Th2 (IL‐4, 5, 13) and Th17 (IL‐17) cytokines. In addition, potential upstream regulators (TSLP, IL‐33, IL‐33 receptor [ST2]) and downstream inflammatory markers of inflammation (IL‐6, CCL11 and Periostin) were quantified in the sera according to manufactures instructions (see Supporting Information S1: Table S1 for the range of each analyte measured in all 708 patients and Supporting Information S1: Table S2 for the maximum detection limit). When protein levels were below the level of detection for specific individuals, these individuals did not contribute to these analyses.

### 2.5 Statistical Analysis

All statistical analyses were carried out on IBM SPSS Statistics 24. See methods in the supplement section for further details on the statistical tests used in this study.

## 3 Results

### 3.1 Demographics and Clinical Features of the Serum Cohort

A total of 708 asthma patients from 18 UK centers were available for analysis as part of the GASP initiative (Table 1). These subjects were enriched with patients meeting GINA 3‐5 (GINA 2018) criteria. Basic demographics, clinical features including spirometry and immunological data were collected, however not all data was available for all 708 patients. Overall patients had poorly controlled asthma with a female predominance (Table 1).

### 3.2 Serum Cytokines Associated With Th2 Inflammation Showed High Coordinated Expression

**Figure 1:**
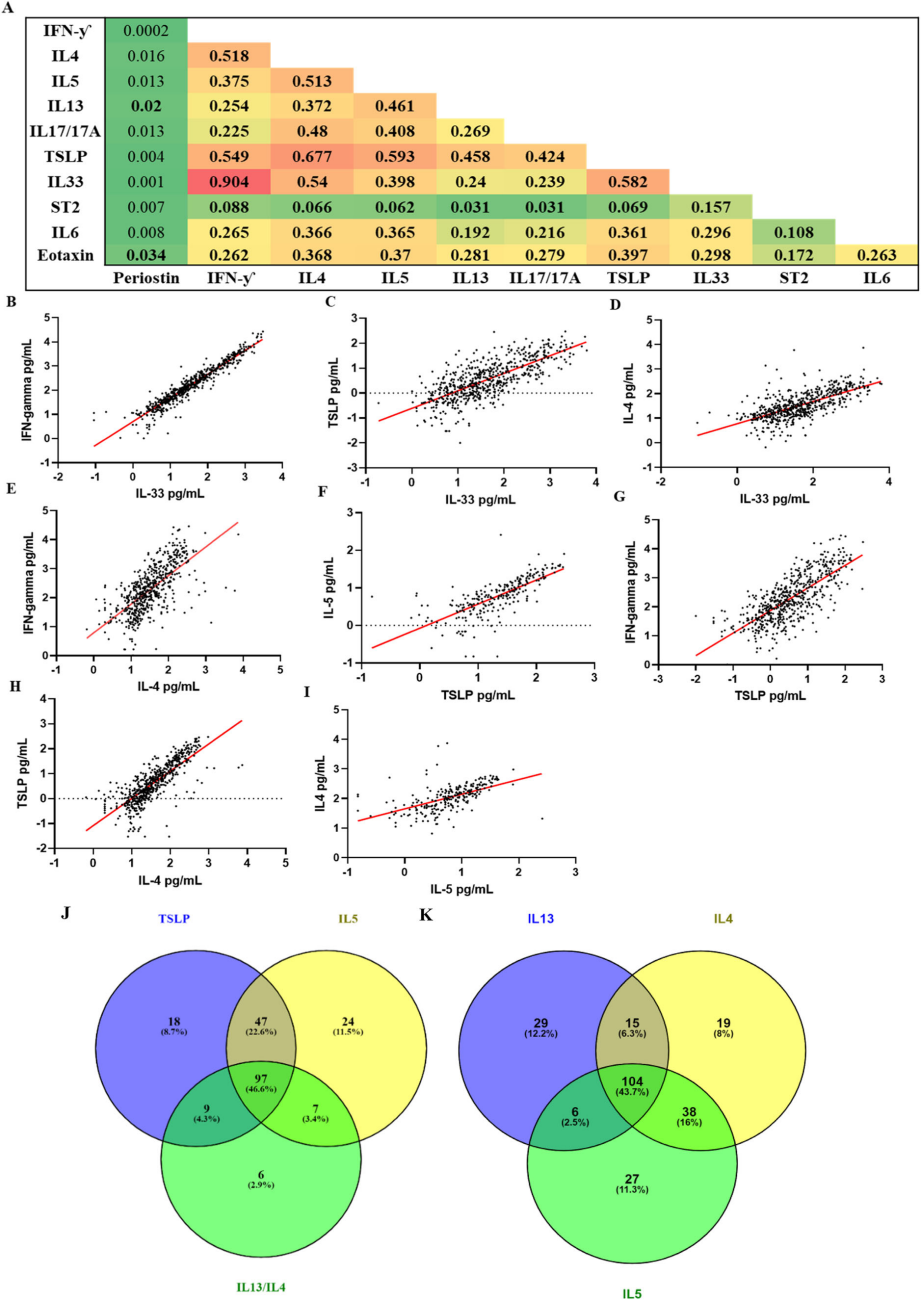
A correlation heat map between each serum cytokine level indicative of Th1 (IFN‐γ), Th2 (IL‐4, IL‐5, IL‐13) and Th17 (IL‐17) and upstream (IL‐33, ST2, TSLP) and downstream (periostin, Eotaxin) markers of inflammatory pathways: (A) *R* ^2^ value is presented for all the correlations between each analyte with red indicating a high *R* ^2^ value and green low. An *R* ^2^ value of > 0.5 was considered biologically significant. *p* values were calculated with spearman correlation and a *p* value of 4 × 10^−^^4^ was considered as statistically significant after Bonferroni correction which are highlighted in bold. *N* = 708 subjects. (B–I) Correlation graphs with line of best fit between serum cytokines that met *R* ^2^ value of > 0.5 and correction, data was plotted as Log_10_. (J–L) Overlap of inflammatory profiles in the serum of asthma patients: (J) patients in the top 25% for cytokines that are current targets of monoclonal antibody drugs (anti‐IL‐5/5 R: Mepolizumab, Reslizumab and Benralizumab, anti‐IL‐4/IL‐13: Dupilumab and anti‐TSLP: Tezepelumab) and their percentage overlap *n* = 208 and (K) patients in the top 25% for the Th2 cytokines (IL‐13, IL‐4 and IL‐5) and their percentage overlap *n* = 238.

Distribution of the serum cytokine data is displayed in Supporting Information S1: Figure S1 and Supporting Information S1: Table S1. Most of the cytokines demonstrated a non‐normal distribution. TSLP showed high correlation with IL‐4, IL‐5 and IFN‐ƴ (*R* ^2^ = 0.68, *R* ^2^ = 0.59, *R* ^2^ = 0.58 and *R* ^2^ = 0.55; respectively) (Figure 1B,F–H). IL‐4 showed high correlation with IL‐33, IL‐5 and IFN‐y (*R* ^2^ = 0.54, *R* ^2^ = 0.513 and *R* ^2^ = 0.52; respectively) (Figure 1D–I). Periostin and ST2 (soluble IL‐33 receptor) showed no correlation with any of the biomarkers.

### 3.3 Cytokines in the Serum Identify a Heterogeneous Inflammatory Profile in Asthma and Suggests Overlapping Inflammatory Profiles

Patients in the top quartile of the three cytokines that reflect current biological targets that are currently in clinic, that is, IL‐5, TSLP, IL‐4/13 (Supporting Information S1: Table S2) demonstrate 46.6% of the asthma patients were high for all three targets (Figure 1J). Whereas Figure 1K demonstrates those patients in the top quartile for the Th2 cytokines (Supporting Information S1: Table S3) with 43.7% of patients being high for all 3 Th2 cytokines.

### 3.4 Childhood Onset Asthma Patients Are More Atopic Than Adult Onset, However, Have a Similar Serum Cytokine Profile

Table 2 shows the demographic, clinical and immunological data stratified by childhood onset < 16 years, early adulthood, 16–34 years, and late adulthood, 35–60 years. The childhood onset group had more atopic comorbidities including atopic dermatitis 32.6% (*p* = 0.001) and allergic rhinitis 63.9% (*p* = 0.001) as well as higher total blood IgE levels 150 KU/L (*p* = 7.8 × 10^−^^8^) compared to both later onset groups (Table 2). Both later age of onset groups demonstrated a higher weight 83.31 kg and 86.12 kg respectively compared to the < 16 group 81.53 kg (*p* = 0.002). Later age of onset groups also demonstrated higher smoking pack years 9 and 10 years respectively (*p* = 1.0 × 10^−^^6^) suggesting early onset patients are less likely to take up smoking in adulthood. FEV_1_ (%Pred) was lower in the < 16 and 16–34 age groups, 80.17% and 79.11% respectively compared to the 35–60 group 83.92% (*p* = 0.003). There was a higher female predominance in the 16–34 group, supporting previous studies that asthma development in middle age has a female bias [20]. No serum cytokine level was associated with age of asthma onset (Supporting Information S1: Table S4).

**Table 2:** Demographics, clinical and immunological features stratified by age of onset: Data are presented as mean and standard deviation (SD), median and minimum and maximum (Min‐Max) or Number (*n*) and percentage (%).

	< 16	16–34	35–60	*p* value
Subjects (*n*)	1090	619	428	—
Age seen (years: Mean and SD)	38.43 (12.07)	44.46 (9.83)	51.28 (5.91)	1.8 × 10^−^^102^
	*n* = 1082	*n* = 614***	*n* = 426***	
Sex (*n*):	1,088	619	428	6.0 × 10^−^^3^
Female	729 (67%)	458 (74%)*	286 (66.8%)	
Height (m: Mean and SD)	1.67 (0.47)	1.66 (0.09)	1.67 (0.47)	7.0 × 10^−^^3^
	*n* = 1019	*n* = 583*	*n* = 410	
Weight (kg: Mean and SD)	81.53 (20.89)	83.31 (20.6)	86.12 (22.62)	2.0 × 10^−^^3^
	*n* = 913	*n* = 507	*n* = 377**	
BMI (kg/m^2^: Median and Min‐Max)	28.04 (14.84–59.52)	29.06 (16.90–78.16)	29.63 (15.82–57.61)	1.15 × 10^−4^
	*n* = 904	*n* = 499*	*n* = 369***	
Smoking status (*n*):	1082	616	427	9.0 × 10^−^^3^
Never	745 (68.9%)	389 (63.1%)	255 (59.7%)	
Ex‐smoker	215 (19.9%)	143 (23.2%)	114 (26.7%)	
Current	122 (11.3%)	84 (13.6%)	58 (13.6%)	
Smoking pack years (Median and Min‐Max)	5.0 (0.04–75)	9 (0.03–161)	10 (0.05–120)	1.0 × 10^−6^
	*n* = 292	*n* = 189**	*n* = 152***	
FEV1(%Pred) (Mean and SD)	80.17 (21.15)	79.11 (23.06)	83.92 (21.4)	3.0 × 10^−3^
	*n* = 944	*n* = 534	*n* = 371*	
FEV_1_FVC (Mean and SD)	0.72 (0.13)	0.72 (0.13)	0.73 (0.11)	1.28 × 10^−^^1^
	*n* = 910	*n* = 513	*n* = 370*	
Change in FEV_1_% reversibility (Median and Min‐Max)	10.24 (−20.12 to 68.75)	9.29 (−18.62 to 61.48)	6.86 (−26.28 to 61.21)	3.34 × 10^−^^1^
	*n* = 273	*n* = 176	*n* = 135	
Peak flow (L/min: Mean and SD)	412.10 (130.68)	383.06 (124.84)	388.07 (136.07)	1.0 × 10^−2^
	*n* = 421	*n* = 256*	*n* = 164*	
Blood total IgE (KU/L: Median and Min‐Max)	150.0 (0.6–4850)	85.50 (1–4190)	89.50 (2–3528)	3.6 × 10^−8^
	*n* = 485	*n* = 288***	*n* = 214***	
Blood Eosinophils (×10^9/L: Median and Min‐Max)	0.23 (0.0–5.15)	0.25 (0.01–5.42)	0.2 (0.1–2.60)	9.85 × 10^−1^
	*n* = 153	*n* = 102	*n* = 77	
Allergic rhinitis (*n*):**	822	457	314	1.0 × 10^−3^
Yes	525 (63.9%)	268 (58.6%)	163 (51.9%)**	
Atopic dermatitis (*n*):**	383	215	143	1.0 × 10^−3^
Yes	125 (32.6%)	47 (21.9%)*	26 (18.3%)**	
Hospital admissions (*n*):	561	286	196	3.72 × 10^−1^
Never	372 (66.3%)	193 (67.5%)	146 (74.5%)	
Once	95 (16.9%)	52 (18.2%)	28 (14.3%)	
More than once	55 (9.8%)	27 (9.4%)	14 (7.1%)	
High dependency/ICU	39 (7.0%)	14 (4.9%)	8 (4.1%)	
Mean ACQ Score 7 (Median and Min‐Max)	1.71 (0–5.83)	1.86 (0–5.71)	1.57 (0–5.43)	1.67 × 10^−1^
	*n* = 521	*n* = 253	*n* = 175	
Mean ACQ Score 6 (Median and Min‐Max)	1.67 (0–6)	1.83 (0–5.67)	1.67 (0–5.33)	1.54 × 10^−^^1^
	*n* = 531	*n* = 263	*n* = 185	
GINA (*n*):	770	424	280	3.39 × 10^−^^1^
1	35 (4.5%)	18 (4.2%)	11 (3.9%)	
2	102 (13.2%)	47 (11.1%)	40 (14.3%)	
3/4	531 (69.0%)	293 (69.1%)	194 (69.3%)	
5	102 (13.2%)	66 (15.6%)	35 (12.5%)	

*Note:* *Age of onset‐ some patients are only known as childhood onset not the actual age so is classed as < 16 therefore, numbers vary for both age of onset variables. **Allergic rhinitis and atopic dermatitis are doctor diagnosis and self‐reported. *p* values were calculated with multinomial logistic regression with < 16 being the reference group * = < 5 × 10^−2^, ** = ≤ 1 × 10^−^^3^ and *** = ≤ 1 × 10^−^^4^. *p* value of ≤ 2 × 10^−^^3^ was considered as statistically significant after Bonferroni correction.

Abbreviations: ACQ, asthma control questionnaire; BMI, body mass index; FEV1 (%Pred), forced expiratory volume in 1 s percentage predicted; FVC, forced vital capacity; GINA, global initiative for asthma; ICU, intensive care unit; IgE, immunoglobulin E.

### 3.5 Patients Prone to Exacerbation Have a Smoking History, Higher BMI, Worse Lung Function and Poor Asthma Symptom Control But Do Not Show Differences in Their Serum Cytokine Profile

Data were first analysed based on patients that did or did not have an exacerbation within the last year (Table 3), then the clinical features that met multiple testing were further analysed based on hospital admission frequency (Supporting Information S1: Table S5). BMI was higher in patients who exacerbated once (29.97 kg/m^2^) or more than once (30.22 kg/m^2^) compared to those who never exacerbated in the previous 12 months (28.61 kg/m^2^) (*p* = 1.0×10^−^^6^) (Table 3). The data showed smoking frequency is highly associated with the risk of an exacerbation and admission to a high dependency/ICU. Smoking pack years was the highest in patients admitted to high dependency/ICU (9 pack years) and high in those who exacerbated once (4.35 pack years) and more than once (4.75 pack years) compared to those that never exacerbated (3.5 pack years) (*p* = 0.0002). FEV_1_ (%Pred) was inversely related to hospital admission frequency (*p* = 8.1 × 10^‐9^). Similarly, the more frequent the hospital admissions the higher the ACQ6 score demonstrating that patients who frequently exacerbate have poor symptom control. Patients with one or more hospital admission or in the high dependency/ICU groups had more severe asthma, GINA 3/4 30.7% and GINA 5 49.2% respectively (*p* = 5.07 × 10^−^^20^), (Supporting Information S1: Figure S2). No serum cytokine level was related to exacerbation prone asthma (Supporting Information S1: Table S6).

**Table 3:** Demographics, clinical and immunological features stratified by hospital admissions: Data are presented as mean & standard deviation (SD), median & minimum and maximum (Min‐Max) or Number (*n*) & percentage (%).

	Never^§^	Ever	*p* value
**Subjects (*n*)**	762	352	—
**Age seen (years: Mean and SD)**	43.40 (11.55)	39.60 (12.33)	7.30 × 10^−^^7^
	*n* = 755	*n* = 347***	
**Age of onset (years: Mean and SD)^†^**	18.60 (16.02)	15.90 (15.03)	1.20 × 10^−^^2^
	*n* = 711	*n* = 332*	
**Age of onset (*n*):^†^**	711	332	1.02 × 10^−^^1^
**< 16** ^**§**^	372 (52.3%)	189 (56.9%)	
**16–34**	193 (27.1%)	93 (28.0%)	
**> 35**	146 (20.5%)	50 (15.1%)*	
**Sex (*n*):**	760	351	2.70 × 10^−^^2^
**Female**	527 (69.3%)	266 (75.8%)*	
**Height (m: Mean and SD)**	1.67 (0.09)	1.66 (0.09)	9.40 × 10^−^^2^
	*n* = 712	*n* = 316	
**Weight (kg: Mean and SD)**	83.02 (20.14)	86.44 (22.57)	1.90 × 10^−^^2^
	*n* = 714	*n* = 309*	
**BMI (Kg/m** ^2^ **: Median and Min‐Max)**	28.66 (14.84–78.16)	29.97 (16.11–57.61)	3.0 × 10^−^^3^
	*n* = 706	*n* = 303*	
**Smoking status (*n*):**	760	351	6.0 × 10^−^^2^
**Never**	586 (77.1%)	252 (71.8%)	
**Ex‐Smoker**	160 (21.1%)	86 (24.5%)	
**Current**	14 (1.8%)	13 (3.7%)*	
**Smoking Pack Years (Median and Min‐Max)**	3.63 (0.03–18)	5 (0.15–52.5)	3.0 × 10^−3^
	*n* = 166	*n* = 90**	
**FEV1(%Pred) (Mean and SD)**	86.46 (20.14)	80.73 (20.51)	3.10 × 10^−^^5^
	*n* = 728	*n* = 316***	
**FEV**_1_ **FVC (Mean and SD)**	0.75 (0.11)	0.74 (0.1)	8.20 × 10^−^^2^
	*n* = 669	*n* = 287	
**Peak Flow (L/min: Mean and SD)**	428.75 (130.37)	408.74 (144.76)	2.16 × 10^−2^
	*n* = 263	*n* = 93	
**Blood total IgE (KU/L: Median and Min‐Max)**	104.0 (2–3528)	167.5 (2–4850)	9.50 × 10^−^^2^
	*n* = 238	*n* = 112	
**Allergic rhinitis (*n*):^‡^**	669	279	1.20 × 10^−^^2^
**Yes**	473 (70.7%)	174 (62.4%)*	
**Atopic dermatitis (*n*):^‡^**	134	65	5.66 × 10^−^^1^
**Yes**	50 (37.3%)	27 (41.5%)	
**Mean ACQ Score 7 (Median and Min‐Max)**	1.57 (0–5.29)	2.42 (0–5.71)	1.52 × 10^−^^12^
	*n* = 648	*n* = 259***	
**Mean ACQ Score 6 (Median and Min‐Max)**	1.5 (0–5.33)	2.33 (0–6)	3.31 × 10^−12^
	*n* = 665	*n* = 269***	
**GINA (*n*):**	733	336	6.25 × 10^−12^
**1/2** ^**§**^	189 (25.8%)	39 (11.6%)	
**3/4**	456 (62.3%)	208 (61.9%)***	
**5**	87 (11.9%)	89 (26.5%)***	

*Note:* †Age of onset‐some patients are only known as childhood onset not the actual age so is marked as < 16 therefore, numbers vary for both age of onset variables. ‡Allergic rhinitis and atopic dermatitis are doctor diagnosis and self‐reported. *p* values were calculated with binomial logistic regression reference groups are indicated with a §. * = < 5 × 10^−^^2^, ** = ≤ 1 × 10^−3^ and *** = ≤ 1 × 10^−4^. *p* value of ≤ 3 × 10^−3^ was considered as statistically significant after Bonferroni correction.

Abbreviations: ACQ, asthma control questionnaire; BMI, body mass index; FEV1 (%Pred), forced expiratory volume in 1 s percentage predicted; FVC, forced vital capacity; GINA, global initiative for asthma; ICU, intensive care unit; IgE, immunoglobulin E.

### 3.6 Asthma Patient Subgroups Defined by Elevated Blood Eosinophil Counts Have Lower Smoking Pack Years and Elevated Total IgE

Asthma patients were stratified based on blood eosinophil count ≥ 300 cells/μL. Patients with elevated eosinophil counts had lower smoking pack years compared to the eosinophil low group (*p* = 0.0003) (Supporting Information S1: Table S7). Total blood IgE levels were higher in the eosinophil high group, 250 KU/L compared to 109 KU/L (*p* = 2.0 × 10^−^^6^).

### 3.7 Two Genetic Signals for Moderate‐Severe Asthma Were Associated With Childhood Onset Asthma

**Figure 2:**
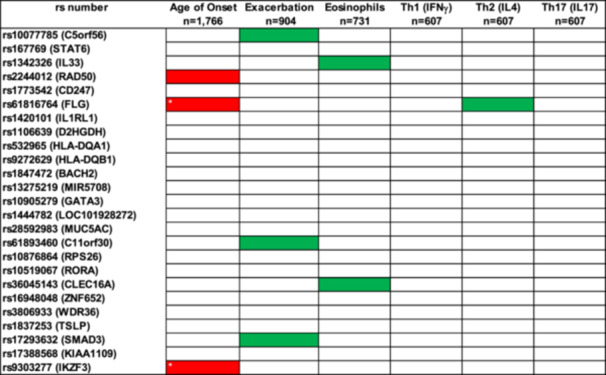
Summary of genetic association data for moderate‐severe risk alleles for clinical features and Th1, Th2 and Th17 serum biomarkers: each square is associated with how the risk allele for that SNP drives the clinical feature or serum biomarker with red meaning it decreases and green it increases. Those in red and green met statistical significance of > 5 × 10^−^^2^ and * met Bonferroni correction of *p* < 2 × 10^−^^3^.

We explored whether carrying 0, 1 or 2 risk alleles for the 25 genetic signals associated with moderate–severe asthma [9] were related with childhood onset, exacerbation prone, eosinophilic high asthma or any of the Th1, 2, 17 biomarkers (Figure 2). Carrying 1 or 2 risk alleles for SNPs rs9303277 (*IKZF3*) and rs61816764 (*FLG*) was associated with childhood onset asthma and met statistical significance after Bonferroni correction. Other genetic signals did show suggestive association with other outcomes but did not meet correction (Supporting Information S1: Tables S8–S11). The genetic risk factors for moderate‐severe asthma did not drive the serum biomarker levels in the blood associated with Th1, Th2 and Th17 inflammation (Supporting Information S1: Tables S12–S14).

## 4 Discussion

This study set out to provide (1) insight into the systemic inflammatory profile in asthma, (2) greater understanding of asthma clinical subgroups and (3) the contribution of genetic risk factors for to (1) and (2).

Nearly 50% of patients overlapped for multiple biomarkers that are current biological targets in clinic that is, IL‐5, TSLP, IL‐4/13. This suggests cytokines may be elevated simultaneously in the same patient and blocking multiple pathways or cytokines may be a suitable strategy for these patients. TSLP was correlated with multiple cytokines including non‐Th2 cytokines suggesting TSLP may be a key upstream regulator [21]. IL‐33 and IFN‐ƴ demonstrated the strongest correlation out of all the cytokines suggesting that the regulation of production of cytokines does not fall into distinct groups such as type 2 or non‐type 2. IL‐33 is also now thought to be an important inducer of Th1 cytokines as IL‐33 has been shown to enhance Th1 responses controlled by IFN‐y in conjunction with IL‐12 [22, 23]. However, we are aware that there has been concern on the validity of serum IL‐33 measurement [9]. A previous study looking at cytokine biomarkers for example, IL‐4, 5, 10, 13, 33 measured from fluid nasal lining and sputum from allergic asthma patients also demonstrated many of the cytokines and chemokines measured (i.e., type‐ 2 cytokines) correlated moderately‐strongly (*r* = > 0.5) with each other (intraorgan comparison). However, when the correlations of these biomarkers between the sputum and nasal fluid were looked at (interorgan comparisons), they showed a moderate‐low relationship (*r* = < 0.5) [16].

Our study represents the largest to date to investigate the utility of characterizing systemic inflammation. However, it did not identify a relationship between the serum inflammatory profiles and any clinical measures and previously described asthma sub‐groups. Additionally, the genetic risk signals associated with moderate‐severe asthma did not influence cytokine levels. These findings suggest that the serum cytokine levels may not be driven by the genetic signals associated with moderate‐severe asthma and are not related to ongoing clinical and immunological features of asthma when stable. Therefore, serum cytokine levels may be poor markers of ongoing disease pathogenesis. Potentially measuring sputum cytokine levels may be a better alternative for studying cytokine profiles in asthma [24].

Previous data has also demonstrated the heterogeneity in airway inflammation amongst asthma patients and using sputum cytokine profiling may be more useful in tailoring asthma therapy by identifying subgroups. Although we cannot conclude if different patient groups based on serum cytokine levels will respond to current biological therapy differently as we did not formally test this. Seys et al. identified 5 asthma subgroups based on sputum cytokine‐high expression (sputum mRNA expression levels of that cytokine were outside the 90th percentile value of the control group). Their data demonstrated that half of the patients had a classical Th2 “high” sputum cytokine profiling. However, when unsupervised cluster analysis was applied, a priori classification of Type‐2 versus non‐Type‐2 molecular subgroup was not identified [18].

### 4.1 Characterization of Asthma Subgroups

Childhood onset is a reproducible subgroup in asthma and highly associated with atopy, high total IgE, prevalence of atopic dermatitis and rhinitis and eosinophilia [20, 25, 26, 27, 28, 29, 30] with the current data further supporting these observations. Late onset patients had a higher BMI, lower lung function, a higher prevalence of smokers and less allergic comorbidities [31, 32, 33], which again was replicated in the current study. Furthermore, signals rs9303277 (*IKZF3*) and rs61816764 (*FLG*) showed the strongest effect size with age of onset when carrying both risk alleles for moderate‐severe asthma compared with all the genetic signals and clinical features. Signals around the *IKZF3* gene have also previously been reported in childhood age of onset GWAS studies [34, 35, 36] suggesting that these signals may drive childhood onset of asthma. A recent study provided evidence for a novel disease‐driving mechanism induced by the 17q21risk allele, which is the strongest genetic risk factor for childhood‐onset asthma. They revealed that increased mucosal GSDMB expression was associated with a cell‐lytic immune response linked with compromised airway immunocompetence. Their findings suggested GSDMB‐related airway cell death and disturbances in the mucosal IFN signature explain the increased vulnerability of 17q21 risk allele carriers to respiratory viral infections in early life. This potentially provides potential novel therapeutic biological targets for interventions in early life with IFN [37]. Overall, these data support distinct clinical and immunological features of these different onset asthma groups [20, 25, 26, 27, 28, 29] and confirm that genetics is important for the risk of developing childhood disease. The systemic inflammatory profile was not different between these groups, again limiting the utility and insight provided.

Exacerbation prone asthma is another important subgroup. We found that higher BMI is associated with more frequent hospital admissions extending data suggesting that BMI is a marker of disease severity in asthma [38]. Poor symptom control and severe asthma defined were also associated with frequent hospital admissions in line with other studies [39, 40, 41]. Patients with frequent exacerbations in the UK severe asthma register also had the strongest association with a high ACQ6 symptom score despite treated with or without OCS [42]. In the current study ACQ6/7 showed a clear association with exacerbation including frequency. We did not identify a specific serum inflammatory profile in these groups and none of the genetic variants associated with the risk of developing moderate‐severe asthma are risk factors for exacerbation in this adult cohort.

We also examined a subgroup characterized by eosinophilia. In our study blood eosinophil count had a weak relationship with IgE driven disease but not with any other clinical distinct subgroups. However, these analyses may have been confounded by the use of corticosteroids, and this may also suggest why elevated eosinophils were specific in identifying distinct asthma subsets. We also demonstrated smoking pack years influenced low blood eosinophil levels. Studies have demonstrated cigarette smoke to influence the immune inflammatory response and can regulate inflammatory mechanisms of eosinophil migration therefore reducing the number of eosinophils [43]. Eosinophil levels in COPD patients have been shown to be lower in the blood suggesting smoking influences eosinophil levels [44]. Again, we did not identify a relationship between systemic inflammation and this asthma subgroup. While not meeting Bonferroni correction, the two nominal genetic associations with rs1342326 (*IL33*), rs36045143 (*CLEC16A*) and blood eosinophil numbers have been described previously [45].

Our study has many strengths; it included well characterized asthma patients with combined used of clinical, genetics and serum biomarker data. Limitations included OCS and ICS use was not well recorded for each patient and so could not be taken into account in the analyses as a covariate. However, in a small subset of patients where we had OCS and ICS data, we looked at whether corticosteroids could influence the levels of these biomarkers with statistically significant effects after Bonferroni correction. A second limitation was some of the biomarkers were difficult to measure in the serum as they fell below the standard curve particularly IL‐5 and IL‐13 this could potentially be because some cytokines have short half‐lives and so a more sensitive assay may be need. In addition, serum is not a direct measurement of airway inflammation and so cytokine levels in sera may not reflect airway inflammation therefore particular cytokines could be lower in the sera than the airways. The population sample was also relatively small for genetic association studies and there was no exhale nitric oxide or sputum eosinophil data recorded as well as limited blood eosinophil data to stratify type‐2 high asthma patients.

## 5 Conclusion

In summary, we have for the first time examined on scale the systemic inflammatory profile in asthma patients, which identified significant heterogeneity with patients presenting with multiple inflammatory profiles. High coordinated expression was seen between most of the Th2 inflammatory biomarkers but also in some of the non‐Th2 biomarkers (IFN‐ƴ) suggesting patients may have their asthma driven by more than one inflammatory pathway. Our data demonstrated that many of these patients had elevated levels of multiple serum cytokines that are targets of newly introduced monoclonal antibodies for asthma. Therefore, these patients could be responsive to a specific and/or combination approaches to treatments, although this is yet to be realized. We confirm and extend previous approaches to characterize and understand asthma subgroups that are clinically relevant. We have provided further evidence that childhood onset disease has an allergic dominance with associated comorbidities and onset in middle age has a female dominance. We also found our genetic data replicated previous associations with childhood onset suggesting signals at the *FLG* and *IKZF3* loci may drive childhood asthma. Similarly, BMI, smoking, lower lung function contributes to the exacerbation prone endotype and that blood eosinophil high patients have elevated total IgE but not accompanying increase in allergic comorbidities.

## Author Contributions

Iain Stewart conceived and designed the study and contributed to the interpretation of the data and writing of the manuscript. Karina Bingham developed and preformed the experiments, analysed the data and wrote the manuscript. Yousef Al Zahrani contributed towards the data input for the database. Michael A. Portelli contributed towards data input of the database and final manuscript. Dominick Shaw contributed towards the supervision and design of the study, interpretation of the data and contribution of final manuscript. All other authors contributed towards collection of patient samples and data, design of the study and final manuscript.

## Ethics Statement

Ethics approvals were either multicentre (MREC‐GM129901) or centre specific. Informed consent was obtained from all participants.

## Conflicts of Interest

The authors declare no conflicts of interest.

## Supporting information

Supporting information.

## Data Availability

The authors have nothing to report.
