# Supplementary material for: Defining the Blood Cytokine Profile in Asthma to Understand Asthma Heterogeneity
Source: Immun Inflamm Dis. 2025 Mar 19;13(3):e70116. doi: 10.1002/iid3.70116 (PMC11920881; doi:10.1002/iid3.70116)
Supplement: Supplementary file 1 — Supporting information. [file IID3-13-e70116-s001.docx]

## Supplement

**Defining the blood cytokine profile in asthma to understand asthma heterogeneity**

Karina Bingham PhD^1^, Yousef Al Zahrani MSc^1,13^, Iain Stewart PhD^2^, Michael A Portelli^1^, PhD^1^, Prof Andrew Fogarty, PhD^3^, Prof Tricia M McKeever, PhD^3^, Ananga Singapuri, BSc^4^, Prof Liam G Heaney, MD^5^, Adel H Mansur, PhD^6^, Prof Rekha Chaudhuri, MD^7^, Prof Neil C Thomson, MD^7^, Prof John W Holloway, PhD^14,15^, Prof Peter H Howarth, PhD^14,15^, Prof Ratko Djukanovic, PhD^14,1^^5^, John D Blakey, PhD^8, 9^, Prof Anoop Chauhan, PhD^10^, Prof Christopher E Brightling^11^, Zara E K Pogson, PhD^12^, Prof Ian P Hall^1^, Prof Luisa Martinez-Pomares PhD^16^, DM, Prof Dominick Shaw DM^1^, Prof Ian Sayers, PhD^1^

^1^Centre for Respiratory Research, NIHR Nottingham Biomedical Research Centre, School of Medicine, Biodiscovery Institute, University of Nottingham, Nottingham, UK.

^2^Faculty of Medicine, National Heart & Lung Institute Imperial College, London, United Kingdom.

^3^Division of Epidemiology and Public Health, University of Nottingham, Nottingham, UK.

^4^Institute for Lung Health, University of Leicester, Glenfield Hospital, Leicester, UK

^5^Centre for Infection and Immunity, Queen’s University of Belfast, Belfast, UK

^6^Respiratory Medicine, Birmingham Heartlands Hospital, Birmingham, UK

^7^Institute of Infection, Immunity and Inflammation, University of Glasgow, Glasgow, UK

^8^Medical School, Curtin University, Perth, Western Australia

^9^Respiratory Medicine, Sir Charles Gairdner Hospital, Perth, WA, Australia

^10^Research and Innovation, Portsmouth Hospitals University NHS Trust, Portsmouth, UK

^11^Department of Health Sciences, University of Leicester, Leicester, UK

^12^United Lincolnshire Hospitals NHS Trust, Lincoln County Hospital, Lincoln, UK

^13^Respiratory Care Department, Prince Sultan Military College of Health Sciences, Dhahran, Kingdom of Saudi Arabia

^14^NIHR Southampton Biomedical Research Centre, University Hospital Southampton, Southampton, UK

^15^Clinical & Experimental Sciences, Faculty of Medicine, University of Southampton, Southampton, UK

^16^School of Life Sciences, Faculty of Medicine and Health Sciences, University of Nottingham, Nottingham, UK

Correspondence

Dr Karina Bingham ([mszkib@exmail.nottingham.ac.uk](mailto:mszkib@exmail.nottingham.ac.uk))

Centre for Respiratory Research, NIHR Nottingham Biomedical Research Centre, School of Medicine, Biodiscovery Institute, University of Nottingham, Nottingham, UK.

**Methods**

**The Genetics of Asthma & Severe Phenotypes (GASP) cohort**

In this study we recruited adult subjects with asthma (age 16-60) from 18 UK centres; Bath (n= 23), Belfast (n= 87), Birmingham (n= 181), Chesterfield (n= 4), Cumbria (n= 315), Derby (n= 52), Glasgow (n= 708), Leicester (n= 123), Lincolnshire (n= 14), Liverpool (n= 17), Manchester (n= 653), Nottingham (n= 821), Northumbria (n= 44), Portsmouth (n= 397), Sheffield (n= 79), Southampton (n= 558), Stoke (n= 126) and West Middlesex (n= 3). Demographics such as Sex, age, age of onset were recorded from each centre along with clinical data including baseline lung function (FEV_1_, FVC and PEF), smoking status, smoking pack years, current asthma medication, GINA (2018) classification and ACQ score. Other desirable measures where available for each patient were also recorded including total IgE levels, number of hospital admissions within the last year, comorbidities such as allergic rhinitis and allergic dermatitis and blood eosinophil counts. Missing data were not imputed.

**Inclusion and exclusion criteria**

Patients eligible for the study were aged 16-60 years, with physician-diagnosed asthma of at least one year duration and current a prescription for asthma medication. Patients recruited had to be of European descent meaning parents and grandparents are both European. Patients were excluded from the study if they had a known highly transmissible or serious blood-borne infection or if they were unable to comprehend the study and provide consent. Patients were excluded if they had a known lung, chest wall, neuromuscular or cardiac disease or abnormality that would confound the Spirometry test, peak flow and the asthma control questionnaire (ACQ) score. Patients with a smoking history of more than ten pack years were excluded from the study. Ethics approvals were either multicentre (MREC-GM129901) or centre specific. Informed consent was obtained from all participants.

**Selecting proxy SNPs for the 25 signals identified in the GWAS**

Patients were genotyped for the 25 genetic signals previously associated with moderate-severe asthma (6). 2,536 subjects used in the current analyses were from this study using UK Biobank array data and so these genotyping data were used were possible. For newly recruited subjects and if the original signal was based on an imputed variant prospective genotyping was completed.

**DNA extraction and genotyping of blood samples**

DNA extraction and genotyping was carried out by an external company LGC Genomics where they used Kompetitive Allele-Specific PCR (KASP) genotyping assay.

**Statistical analysis**

To identify whether continuous variables were normally distributed, histograms were created to determine distribution. Normally distributed data were summarised using mean with standard deviation or median with the range. Non-normally distributed data were log-transformed or square root prior to association testing. Missing data was not imputed. A general or multinomial logistic model or chi squared test was used for categorical variables. Student’s t-test or Mann-Whitney U test for two independent samples, a one-way ANOVA or a Kruskal-Wallis test for more than two independent samples or univariate model when considering covariates were used for continuous data. R^2^ values were calculated using Spearman's rank correlation for non-normally distributed data or Pearson’s correlation coefficient for normally distributed data. Adjustment for multiple testing was applied using Bonferroni correction. Centre heterogeneity could not be considered in statistically analysis due to some centres only having less than 5 patients used in the analysis. For most of the genetics and clinical analysis two sets of analysis was carried out. The first analysis to look at the differences between the different risk allele groups (0, 1, 2). The second statistical test to see where the direction of effect was coming from and to also consider any cofounders based on those genetic signals that met statistical significance of p= >0.05. Post hoc power analysis on the genetic data was also carried out on SPSS or G*power on the genetic data to aid interpretation of the results and to calculate power.

| Analyte | Total number of patients with a measurement | Median  (pg/mL) | Min-max  (pg/mL) |
| --- | --- | --- | --- |
| IFN-gamma | 652 | 135.76 | 1.02-29,189.50 |
| IL-4 | 631 | 29.67 | 0.65-7,406.05 |
| IL-5 | 247 | 6.08 | 0.15-257.93 |
| IL-13 | 324 | 362.33 | 1.20-8,050.07 |
| IL17/17A | 438 | 3.70 | 0.01-952.43 |
| TSLP | 642 | 3.20 | 0.01-298.99 |
| IL-33 | 674 | 27.34 | 0.09-6,238.30 |
| ST2 | 699 | 9,544.19 | 955.62-4,010,000.0 |
| IL-6 | 698 | 1.94 | 0.01-178.55 |
| Eotaxin | 697 | 165.38 | 4.67-2,481.32 |
| Periostin | 708 | 7,7536.63 | 9,219.04-699,421.52 |

**Supplementary Table 1 Total number of patients with measurable serum cytokine levels for each analyte out of 708 asthma patients:** Data is displayed as the median and the minimum-maximum (min-max) for each analyte in those subject that fell within the standard range. Some of the higher concentrations were extrapolated from the standard curve.

| Analyte | Maximum detection limit pg/mL |
| --- | --- |
| Eotaxin | 14,750 |
| IL-4 | 3,380 |
| IL-6 | 1,400 |
| IL17/17A | 2,870 |
| Periostin | 351,020 |
| TSLP | 710 |
| IFN-gamma | 12,330 |
| IL-5 | 1,440 |
| IL-13 | 95,060 |
| IL-33 | 2,660 |
| ST2 | 95,470 |

**Supplementary Table 2 Luminex maximum detection limit for each analyte:** a maximum concentration was entered during the standard setup on the bioplex 200 machine along with a dilution fold of 3.

**Supplementary Figure 1 Violin plots showing the distribution of the serum cytokine data:** thick dotted line displays the median and the small, dotted lines display the quartiles. Those with extremely high values were omitted from the violin plots to aid visualisation but retained in the analyses. Many of the patients Th2 cytokines measurements fell below the standard curve, for the analysis they were given a measurement of zero.

| Biomarker | Top 25% lowest cut off point |
| --- | --- |
| IL-4 | ≥75.51 pg/mL |
| IL-5 | ≥3.48 pg/mL |
| IL-13 | ≥398.72 pg/mL |
| TSLP | ≥11.54 pg/mL |

**Supplementary Table 3 Cytokine lowest cut off value for each top quartile group**

|  | <16 | 16-34 | >35 | P value |
| --- | --- | --- | --- | --- |
| Subjects (n) | 369 | 176 | 122 | - |
| IFN-y (log_10_ pg/mL) | 2.24 (0.82)  N= 344 | 2.16 (0.80)  N= 163 | 2.20 (0.85)  N= 105 | 5.76 x10^-1^ |
| IL-4 (log_10_ pg/mL) ^‡^ | 1.56 (0.58)  N= 334 | 1.51 (0.54)  N= 160 | 1.62 (0.62)  N= 100 | 4.22 x10^-1^ |
| IL-5 (log_10_ pg/mL) | 0.72 (0.48)  N= 138 | 0.84 (0.56)  N= 56 | 0.81 (0.51)  N= 43 | 2.72 x10^-1^ |
| IL-13 (log_10_ pg/mL) ^‡^ | 2.58 (0.46)  N= 177 | 2.48 (0.58)  N= 78 | 2.71 (0.50)  N= 52 | 7.0 x10^-2^ |
| IL17/17A (log_10_ pg/mL) | 0.60 (0.80)  N= 237 | 0.61 (0.74)  N= 102 | 0.77 (0.84)  N= 79 | 2.58 x10^-1^ |
| TSLP (log_10_ pg/mL) ^‡^ | 0.58 (0.75)  N= 343 | 0.54 (0.82)  N= 160 | 0.62 (0.93)  N= 100 | 8.26 x10^-1^ |
| IL-33 (log_10_ pg/mL) | 1.60 (0.83)  N= 354 | 1.48 (0.79)  N= 168 | 1.48 (0.93)  N= 113 | 1.89 x10^-1^ |
| ST2 (log_10_ pg/mL) ^†‡^ | 4.0 (0.36)  N= 359 | 4.04 (0.40)  N= 172 | 4.04 (0.42)  N= 119 | 5.66 x10^-1^ |
| IL-6 (log_10_ pg/mL) ^†^ | 0.29 (0.47)  N= 359 | 0.31 (0.44)  N= 171 | 0.35 (0.52)  N= 121 | 9.99 x10^-1^ |
| Eotaxin (log_10_ pg/mL) ^†‡^ | 2.20 (0.32)  N= 359 | 2.23 (0.32)  N= 172 | 2.24 (0.35)  N= 118 | 8.02 x10^-1^ |
| Periostin (log_10_ pg/mL) ^‡^ | 4.87 (0.16)  N= 367 | 4.88 (0.15)  N= 176 | 4.88 (0.19)  N= 122 | 9.25 x10^-1^ |

**Supplementary Table 4 Serum Cytokine Data Stratified Based on Age of Onset n= 667:** Data is presented as mean & standard deviation log_10_ pg/mL A univariate analysis was used to analyse the data and biomarker indicated with ^†^ were adjusted for age and/or ^‡^ were adjusted for Sex.

|  | Never | Once | More than once | High dependency/ ICU | P-Value |
| --- | --- | --- | --- | --- | --- |
| Subjects (n) | 762 | 185 | 104 | 63 | - |
| BMI (Kg/m^2^: (Median & Min-Max) | 28.64 (14.84-78.16)  (n= 702) | 29.97 (16.11-57.61)  (n= 161) ** | 30.22 (19.38-55.17)  (n= 87) * | 29.40 (16.85-54.90)  (n= 53) | **1.0x10^-6^** |
| Smoking pack years | 3.5 (0.03-18)  (n= 165) | 4.35 (0.5-35)  (n= 43) * | 4.75 (0.15-52.5)  (n= 30) * | 9 (0.5-39)  (n= 17) * | **2.0x10^-4^** |
| FEV1(%Pred) (Mean & S.D) | 86.46 (20.14)  (n= 723) | 82.62 (1.35)  (n= 164) * | 79.98 (22.08)  (n= 95) ** | 76.14 (19.92)  (n= 53) *** | **8.1x10^-9^** |
| Mean ACQ Score 6 (Mean & S.D) | 1.73 (1.25)  (n= 665) | 2.24 (1.35)  (n= 154) | 2.65 (1.46)  (n= 73) | 3.01 (1.57)  (n= 42) | **9.63x10^-16^** |
| GINA (n):  1/2  3/4  5 | 726  189 (26%)  452 (62.3%)  85 (11.7%) | 169  26 (15.4%) **  115 (68%)  28 (16.6%) | 101  9 (8.9%) ***  61 (60.4%) ***  31 (30.7%) | 61  4 (6.6%) ***  27 (44.2%) ***  30 (49.2%) | **5.07x10^-20^** |

**Supplementary Table 5: Clinical features of asthma patients stratified by hospital admission frequency:** Data are presented as mean & standard deviation (S.D), median & minimum and maximum (Min-Max) or Number (n) & percentage (%). BMI: body mass index; FEV1(%Pred): forced expiratory volume in 1 s percentage predicted; ICU: intensive care unit; ACQ: asthma control questionnaire; GINA: global initiative for asthma (2018). P-values were calculated with multinomial logistic regression and corrected for age and never being the reference group *= <5x10^-2^, **= ≤1x10^-3^ and ***= ≤1x10^-4^. P value of ≤8x10^-3^ was considered as statistically significant after Bonferroni correction.

**Supplementary Figure 2 Exacerbation frequency across asthma severity based on GINA 2018**: GINA 5 patients show a significant increase in hospital admission frequency compared to those patients in GINA 1/2 and 3/4.

|  | Never | Ever | P Value |
| --- | --- | --- | --- |
| Subjects (n) | 407 | 234 | - |
| IFN-y (log_10_ pg/mL) | 2.22 (0.82)  N= 375 | 2.16 (0.83)  N= 220 | 3.64x10^-1^ |
| IL-4 (log_10_ pg/mL) ^‡^ | 1.58 (0.58)  N= 360 | 1.52 (0.56)  N= 206 | 2.74x10^-1^ |
| IL-5 (log_10_ pg/mL) | 0.74 (0.50)  N= 148 | 0.77 (0.51)  N= 80 | 7.04x10^-1^ |
| IL-13 (log_10_ pg/mL) ^‡^ | 2.59 (0.53)  N= 193 | 2.53 (0.46)  N= 106 | 4.04x10^-1^ |
| IL17/17A (log_10_ pg/mL) | 0.64 (0.82)  N= 265 | 0.59 (0.72)  N= 142 | 5.53x10^-1^ |
| TSLP (log_10_ pg/mL) ^‡^ | 0.59 (0.79)  N= 373 | 0.55 (0.81)  N= 151 | 6.39x10^-1^ |
| IL-33 (log_10_ pg/mL) | 1.54 (0.86)  N= 394 | 1.48 (0.80)  N= 218 | 3.94 x10^-1^ |
| ST2 (log_10_ pg/mL) ^†‡^ | 4.0 (0.36)  N= 399 | 4.01 (0.38)  N= 225 | 3.03 x10^-1^ |
| IL-6 (log_10_ pg/mL) ^†^ | 0.28 (0.50)  N= 398 | 0.32 (0.44)  N= 228 | 1.62x10^-1^ |
| Eotaxin (log_10_ pg/mL) ^†‡^ | 2.23 (0.32)  N= 396 | 2.19 (0.35)  N= 228 | 5.26x10^-1^ |
| Periostin (log_10_ pg/mL) ^‡^ | 4.88 (0.15)  N= 406 | 4.86 (0.18)  N= 233 | 2.32x10^-1^ |

**Supplementary Table 6 Serum Biomarker Data Stratified Based on Hospital Admissions:** n= 641 Data is presented as mean & standard deviation log10 pg/mL A univariate analysis was used to analyse the data and biomarker indicated with † were adjusted for age and/or ‡ were adjusted for Sex.

|  | **<300 cells/µL** | **>300 cells/µL** | **P Value** |
| --- | --- | --- | --- |
| **Subjects (n)** | 507 | 304 | - |
| **Age seen (years: Mean & S.D)** | 45.07 (10.87)  (n= 505) | 44.38 (11.34)  (n= 303) | 3.9x10^-1^ |
| ***Age of onset (years: Mean & S.D)** | 21.30 (15.91)  (n= 196) | 19.48 (16.40)  (n= 138) | 3.09x10^-1^ |
| ***Age of onset (n):**  **<16**  **16-34**  **>35** | 196  90 (45.9%)  58 (29.6%)  48 (24.5%) | 138  65 (47.1%)  44 (31.9%)  29 (21%) | 7.45x10^-1^ |
| **Sex (n):**  **Female** | 507  353 (69.6%) | 304  197 (64.8%) | 1.55x10^-1^ |
| **Height (m: Mean & S.D)** | 1.65 (0.09)  (n= 459) | 1.66 (0.1)  (n= 267) | 1.46x10^-1^ |
| **Weight (kg: Mean & S.D)** | 79.57 (20.47)  (n= 460) | 80.5 (21.75)  (n= 266) | 5.63x10^-1^ |
| **BMI (Kg/m^2^: Median & Min-Max)** | 28.17 (15.41-51.53)  (n= 456) | 27.52 (17.78-60.24)  (n= 264) | 7.01x10^-1^ |
| **Smoking status (n):**  **Never**  **Ex-Smoker**  **Current** | 503  276 (54.9%)  121 (24.1%)  106 (21.1%) | 303  166 (54.8%)  85 (28.1%)  52 (17.2%) | 2.65x10^-1^ |
| **Smoking Pack Years (Median & Min-Max)** | 20.0 (0.04-108)  (n= 110) | 9.25 (0.05-120)  (n= 70) | **3.0 x10^-4^** |
| **FEV1(%Pred) (Mean & S.D)** | 75.42 (24.11)  (n= 424) | 71.59 (22.57)  (n= 219) | 5.2 x10^-2^ |
| **FEV_1_FVC (Mean & S.D)** | 0.67 (0.14)  (n= 469) | 0.66 (0.13)  (n= 279) | 2.72x10^-1^ |
| **Change in FEV_1_% Reversibility (Median & Min-Max)** | 7.86 (-8.79-61.48)  (n= 149) | 8.17 (-26.28-68.75)  (n= 114) | 3.74x10^-1^ |
| **Peak Flow (L/min: Mean & S.D)** | 361.74 (126.41)  (n= 374) | 362 (127.8)  (n= 176) | 9.82x10^-1^ |
| **Blood total IgE (KU/L: Median & Min-Max)** | 109.0 (1-4,900)  (n= 390) | 250.0 (1.66-4,700)  (n= 245) | **2.0x10^-6^** |
| ****Allergic rhinitis (n):**  **Yes** | 223  108 (48.4%) | 162  89 (54.9%) | 2.07x10^-1^ |
| ****Atopic dermatitis (n):**  **Yes** | 193  68 (35.2%) | 93  30 (32.3%) | 6.19x10^-1^ |

**Table 7 Demographics, clinical and immunological features stratified on the blood eosinophil count:** Data are presented as mean & standard deviation (S.D), median & minimum and maximum (Min-Max) or Number (n) & percentage (%). BMI: body mass index; FEV1(%Pred): forced expiratory volume in 1 s percentage predicted; FVC: forced vital capacity; IgE: immunoglobulin E. * Age of onset- some patients are only known as childhood onset not the actual age so is marked as <16 therefore, numbers vary for both age of onset variables. **Allergic rhinitis and atopic dermatitis are doctor diagnosis and self-reported. P-values were calculated with binomial logistic regression with <300 cells/µL being the reference group *= <5x10^-2^, **= ≤1x10^-3^ and ***= ≤1x10^-4^. P value of ≤3x10^-3^ was classed as statistically significant after Bonferroni correction.

| Rs number | 0 | 1 | 2 | P-value |
| --- | --- | --- | --- | --- |
| rs10077785  (*C5orf56*) | N= 976  <16: 484 (49.6%)  16-34: 291 (29.8%)  35-60: 201 (20.6%) | N= 651  <16: 340 (52.2%)  16-34: 180 (27.6%)  35-60: 131 (20.1%) | N= 131  <16: 67 (51.1%)  16-34: 30 (29.8%)  35-60: 25 (19.1%) | 8.53x10^-1^ |
| rs167769  (*STAT6*) | N= 582  <16: 284 (48.8%)  16-34: 158 (27.1%)  35-60: 140 (24.1%) | N= 861  <16: 438 (50.9%)  16-34: 259 (301%)  35-60: 164 (19%) | N= 313  <16: 171 (54.6%)  16-34: 90 (28.8%)  35-60: 52 (16.6%) | 5.6x10^-2^ |
| rs1342326  (*IL33*) | N= 1,128  <16: 558 (49.5%)  16-34: 339 (30.1%)  35-60: 231 (20.5%) | N= 566  <16: 302 (53.4%)  16-34: 155 (27.4%)  35-60: 109 (19.3%) | N= 61  <16: 30 (49.2%)  16-34: 15 (24.6%)  35-60: 16 (26.2%) | 4.22x10^-1^ |
| rs2244012  (*RAD50*) | N= 918  <16: 429 (46.7%)  16-34: 293 (31.9%)  35-60: 196 (21.4%) | N= 474  <16: 256 (54%)  16-34: 125 (26.4%)  35-60: 93 (19.6%) | N= 76  <16: 38 (50%)  16-34: 28 (36.8%)  35-60: 10 (13.2%) | **3.8x10^-2^** |
| rs1773542  (*CD247*) | N= 291  <16: 140 (48.1%)  16-34: 87 (29.9%)  35-60: 64 (22%) | N= 803  <16: 407 (50.7%)  16-34: 235 (29.3%)  35-60: 161 (20%) | N= 660  <16: 343 (52%)  16-34: 186 (28.2%)  35-60: 131 (19.8%) | 8.53x10^-1^ |
| rs61816764  (FLG) | N= 1,544  <16: 756 (49%)  16-34: 463 (30%)  35-60: 325 (21%) | N= 211  <16: 136 (64.5%)  16-34: 45 (21.3%)  35-60: 30 (14.2%) | | **1.0x10^-4^** |
| rs1420101  (*IL1RL1*) | N= 462  <16: 224 (48.5%)  16-34: 146 (31.6%)  35-60: 92 (19.9%) | N= 824  <16: 414 (50.2%)  16-34: 235 (28.5%)  35-60: 175 (21.2%) | N= 465  <16: 250 (53.8%)  16-34: 128 (27.5%)  35-60: 87 (18.7%) | 4.34x10^-1^ |
| rs1106639  (*D2HGDH*) | N= 84  <16: 46 (54.8%)  16-34: 21 (25%)  35-60: 17 (20.2%) | N= 624  <16: 304 (48.7%)  16-34: 187 (30%)  35-60: 133 (21.3%) | N= 1,046  <16: 541 (51.7%)  16-34: 300 (28.7%)  35-60: 205 (19.6%) | 6.92x10^-1^ |
| rs532965  (*HLA-DQA1*) | N= 1,018  <16: 510 (50.1%)  16-34: 296 (29.1%)  35-60: 212 (20.8%) | N= 656  <16: 331 (50.5%)  16-34: 198 (30.2%)  35-60: 127 (19.4%) | N= 77  <16: 45 (58.4%)  16-34: 16 (20.8%)  35-60: 16 (20.8%) | 4.75x10^-1^ |
| rs9272629  (*HLA-DQB1*) | N= 284  <16: 133 (46.8%)  16-34: 76 (26.8%)  35-60: 75 (26.4%) | N= 554  <16: 289 (52.2%)  16-34: 160 (28.9%)  35-60: 105 (19%) | N= 645  <16: 187 (46.6%)  16-34: 133 (33.2%)  35-60: 81 (20.2%) | 3.8x10^-1^ |
| rs1847472  (*BACH2*) | N= 197  <16: 107 (54.3%)  16-34: 58 (29.4%)  35-60: 32 (16.2%) | N= 788  <16: 388 (49.2%)  16-34: 235 (29.8%)  35-60: 165 (20.9%) | N= 768  <16: 337 (52.2%)  16-34: 189 (29.3%)  35-60: 119 (18.4%) | 7.2x10^-2^ |
| rs13275219  (*MIR5708*) | N= 699  <16: 359 (51.4%)  16-34: 197 (28.2%)  35-60: 143 (20.5%) | N= 828  <16: 409 (49.4%)  16-34: 250 (30.2%)  35-60: 169 (20.4%) | N= 226  <16: 123 (54.4%)  16-34: 60 (26.5%)  35-60: 43 (19%) | 7.04x10^-1^ |
| rs10905279  (*GATA3*) | N= 604  <16: 330 (54.6%)  16-34: 155 (25.7%)  35-60: 119 (19.7%) | N= 886  <16: 429 (48.4%)  16-34: 273 (30.8%)  35-60: 184 (20.8%) | N= 266  <16: 132 (49.6%)  16-34:81 (30.5%)  35-60: 53 (19.9%) | 1.62x10^-1^ |
| rs1444782  (*LOC101928272*) | N= 257  <16: 138 (53.7%)  16-34: 74 (28.8%)  35-60: 45 (17.5%) | N= 850  <16: 440 (51.8%)  16-34: 234 (27.5%)  35-60: 176 (20.6%) | N= 646  <16: 312 (48.3%)  16-34: 199 (30.8%)  35-60: 135 (20.9%) | 4.19x10^-1^ |
| rs28592983  (*MUC5AC*) | N= 746  <16: 390 (52.3%)  16-34: 200 (26.8%)  35-60: 156 (20.9%) | N= 761  <16: 381 (50.1%)  16-34: 238 (31.3%)  35-60: 142 (18.7%) | N= 244  <16: 116 (47.5%)  16-34: 71 (29.1%)  35-60: 57 (23.4%) | 2.17x10^-1^ |
| rs61893460  (*C11orf30*) | N= 468  <16: 217 (46.4%)  16-34: 155 (33.1%)  35-60: 96 (20.5%) | N= 834  <16: 436 (52.3%)  16-34: 226 (27.1%)  35-60: 172 (20.6%) | N= 444  <16: 232 (52.3%)  16-34: 126 (28.4%)  35-60: 86 (19.4%) | 1.69x10^-1^ |
| rs10876864  (*RPS26*) | N= 384  <16: 201 (52.3%)  16-34: 111 (28.9%)  35-60: 72 (18.8%) | N= 552  <16: 277 (50.2%)  16-34: 161 (29.2%)  35-60: 114 (20.7%) | N= 342  <16: 174 (50.9%)  16-34: 101 (29.5%)  35-60: 67 (19.6%) | 9.93x10^-1^ |
| rs10519067  (*RORA*) | N= 373  <16: 184 (49.3%)  16-34: 96 (25.7%)  35-60: 93 (24.9%) | | N= 1,387  <16: 710 (51.2%)  16-34: 413 (29.8%)  35-60: 264 (19%) | **3.2 x10^-2^** |
| rs36045143  (*CLEC16A*) | N= 111  <16: 50 (45%)  16-34: 33 (29.7%)  35-60: 28 (25.2%) | N= 597  <16: 301 (50.4%)  16-34: 173 (29%)  35-60: 123 (20.6%) | N= 1,050  <16: 541 (51.5%)  16-34: 304 (29%)  35-60: 205 (19.5%) | 6.38x10^-1^ |
| rs16948048  (*ZNF652*) | N= 665  <16: 313 (47.1%)  16-34: 202 (30.4%)  35-60: 150 (22.6%) | N= 834  <16: 439 (52.6%)  16-34: 227 (27.2%)  35-60: 168 (20.1%) | N= 255  <16: 137 (53.7%)  16-34: 80 (31.4%)  35-60: 38 (14.9%) | **4.1x10^-2^** |
| rs3806933  (*WDR36*) | N= 301  <16: 158 (52.5%)  16-34: 87 (28.9%)  35-60: 56 (18.6%) | N= 822  <16: 411 (50%)  16-34: 238 (29%)  35-60: 173 (21%) | N= 635  <16: 323 (50.9%)  16-34: 185 (29.1%)  35-60: 127 (20%) | 9.16x10^-1^ |
| rs1837253  (*TSLP*) | N= 89  <16: 45 (50.6%)  16-34: 24 (27%)  35-60: 20 (22.5%) | N= 613  <16: 306 (49.9%)  16-34: 187 (30.5%)  35-60: 120 (19.6%) | N= 1,058  <16: 543 (51.3%)  16-34: 299 (28.3%)  35-60: 216 (20.4%) | 8.57x10^-1^ |
| rs17293632  (*SMAD3*) | N= 947  <16: 477 (50.4%)  16-34: 268 (28.3%)  35-60: 202 (21.3%) | N= 707  <16: 350 (49.5%)  16-34: 219 (31%)  138 (19.5%) | N= 104  <16: 64 (61.5%)  16-34: 24 (23.1%)  35-60: 16 (15.4%) | 1.39 x10^-1^ |
| rs17388568  (*KIAA1109*) | N= 896  <16: 459 (51.2%)  16-34: 240 (26.8%)  35-60: 197 (22%) | N= 723  <16: 369 (51%)  16-34: 228 (31.5%)  35-60: 126 (17.4%) | N= 130  <16: 59 (45.4%)  16-34: 38 (29.2%)  35-60: 33 (25.4%) | **4.5 x10^-2^** |
| rs9303277  (*IKZF3*) | N= 414  <16: 168 (40.6%)  16-34: 132 (31.9%)  35-60: 114 (27.5%) | N= 822  <16: 430 (52.3%)  16-34: 241 (29.3%)  35-60: 151 (18.4%) | N= 479  <16: 270 (56.4%)  16-34: 125 (26.1%)  35-60: 84 (17.5%) | **1.0x10^-5^** |

**Supplementary Table 8 Genetic signals identified in the moderate to severe asthma GWAS and their association with age of onset:** data is presented as percentage and numbers between the age of onset groups across the risk allele groups for moderate-severe asthma with 0 indicating carrying no risk alleles for moderate-severe asthma, 1 carrying 1 risk allele for moderate-severe asthma and 2 carrying 2 risk alleles for moderate-severe asthma. Chi-squared test was used to analyse both these categorical groups. *Two genotyping groups were combined due to the subject numbers being low in one of the groups. Bonferroni correction p= <2x10^-3^.

| Age of onset | | | | | |
| --- | --- | --- | --- | --- | --- |
| Locus | **0** | **1** | **2** | **P-value** | **Power** |
| FLG  (rs61816764) | 19.20 (S.D 15.63)  N= 1,544 | 15.02 (S.D 15.18)  N= 211 | | **2.67x10^-4^** | 1.0 |
| IKZF3  (rs9303277) | 22.32 (S.D 15.82)  N= 414 | 18.20 (S.D 15.36)  N= 822 | 16.75 (S.D 15.46)  N= 479 | **2.20x10^-7^** | 1.0 |

**Supplementary Table 9 FLG and IKZF3 signals showed association with age of onset:** data is presented as mean and standard deviation across the risk allele groups for moderate-severe asthma with 0 indicating carrying no risk alleles for moderate-severe asthma, 1 carrying 1 risk allele for moderate-severe asthma and 2 carrying 2 risk alleles for moderate-severe asthma. One-ANOVA or an independent T-test was used to analyse the age of onset as a linear variable with the genetic signals. *Two genotyping groups were combined due to the n’s being low in one of the groups. Some of the signals from the previous analysis in 6.4.3A however did not meet Bonferroni correction p= <8x10^-3^.

| Rs number | 0 | 1 | 2 | P-value |
| --- | --- | --- | --- | --- |
| rs10077785  (*C5orf56*) | N= 496  153 (30.7%) | N= 330  117 (34.9%) | N= 63  30 (47.6%) | **2.0x10^-2^** |
| rs167769  (*STAT6*) | N= 279  98 (35%) | N= 441  149 (33.4%) | N= 169  53 (31.2%) | 5.86x10^-1^ |
| rs1342326  (*IL33*) | N= 576  191 (32.9%) | N= 286  97 (33.7%) | N= 25  8 (30.8%) | 9.01x10^-1^ |
| rs2244012  (*RAD50*) | N= 533  176 (32.8%) | N= 317  110 (34.4%) | N= 37  13 (35.1%) | 09.46x10^-1^ |
| rs1773542  (*CD247*) | N= 149  49 (32.7%) | N= 400  140 (34.9%) | N= 337  108 (31.6%) | 5.74x10^-1^ |
| rs61816764  (FLG) | N= 783  264 (33.7%) | N= 113  35 (31%) | | 5.63x10^-1^ |
| rs1420101  (*IL1RL1*) | N= 152  52 (34%) | N= 409  142 (34.6%) | N= 326  (31.4%) | 6.86x10^-1^ |
| rs1106639  (*D2HGDH*) | N= 46  12 (26.1%) | N= 296  99 (33.2%) | N= 544  188 (34.2%) | 5.76x10^-1^ |
| rs532965  (*HLA-DQA1*) | N= 531  172 (32.1% | N= 314  106 (33.7%) | N= 42  19 (44.2%) | 1.98x10^-1^ |
| rs9272629  (*HLA-DQB1*) | N= 185  58 (31.4%) | N= 281  94 (33.1%) | N= 385  (34.4%) | 7.98x10^-1^ |
| rs1847472  (*BACH2*) | N= 93  34 (36.2%) | N= 405  134 (32.8%) | N= 389  130 (33.2%) | 8.58x10^-1^ |
| rs13275219  (*MIR5708*) | N= 354  125 (35%) | N= 417  143 (34%) | N= 114  29 (25.5%) | 9.1x10^-2^ |
| rs10905279  (*GATA3*) | N= 328  109 (33%) | N= 434  141 (32.2%) | N= 126  49 (38.3%) | 5.29x10^-1^ |
| rs1444782  (*LOC101928272*) | N= 109  38 (34.8%) | N= 442  145 (32.4%) | N= 334  115 (34.3%) | 7.17x10^-1^ |
| rs28592983  (*MUC5AC*) | N= 392  123 (31.2%) | N= 385  130 (33.3%) | N= 107  44 (41.1%) | 1.29x10^-1^ |
| rs61893460  (*C11orf30*) | N= 209  59 (27.7%) | N= 549  173 (29.8%) | N= 220  66 (29.7%) | **6.0x10^-3^** |
| rs10876864  (*RPS26*) | N= 267  87 (32.1%) | N= 455  153 (33.5%) | N= 164  59 (35.8%) | 6.92x10^-1^ |
| rs10519067  (*RORA*) | N= 169  46 (27.2%) | | N= 729  253 (34.7%) | 6.3x10^-2^ |
| rs36045143  (*CLEC16A*) | N= 55  18 (32.7%) | N= 321  96 (29.6%) | N= 516  186 (35.8%) | 2.99x10^-1^ |
| rs16948048  (*ZNF652*) | N= 317  108 (33.8%) | N= 438  152 (34.5%) | N= 130  36 (27.3%) | 3.12x10^-1^ |
| rs3806933  (*WDR36*) | N= 143  42 (29.2%) | N= 417  141 (33.7%) | N= 330  116 (34.7%) | 4.68x10^-1^ |
| rs1837253  (*TSLP*) | N= 47  19 (40.4%) | N= 310  98 (31.2%) | N= 535  182 (33.8%) | 3.28x10^-1^ |
| rs17293632  (*SMAD3*) | N= 480  153 (31.5%) | N= 356  117 (32.8%) | N= 53  27 (50%) | **2.0x10^-2^** |
| rs17388568  (*KIAA1109*) | N= 414  139 (33.2%) | N= 395  133 (33.5%) | N= 75  24 (32%) | 9.47x10^-1^ |
| rs9303277  (*IKZF3*) | N= 197  64 (32.3%) | N= 404  134 (32.9%) | N= 247  86 (34.4%) | 9.39x10^-1^ |

**Supplementary Table 10 Genetic signals identified in the moderate to severe asthma GWAS and their association with hospital admissions:** data is presented as percentage and N’s for the patients who had a hospital admission in the last year and the total number of patients who had hospital admissions data across the risk allele groups for moderate-severe asthma with 0 indicating carrying no risk alleles for moderate-severe asthma, 1 carrying 1 risk allele for moderate-severe asthma and 2 carrying 2 risk alleles for moderate-severe asthma.. A chi-squared test was used to analyse these categorical groups. Bonferroni correction p= <2x10^-3^.

| Rs number | 0 | 1 | 2 | P-value |
| --- | --- | --- | --- | --- |
| rs10077785  (*C5orf56*) | N= 360  137 (38.1%) | N= 310  111 (35.8%) | N= 61  27 (44.3%) | 4.47x10^-1^ |
| rs167769  (*STAT6*) | N= 240  96 (40%) | N= 347  129 (37.2%) | N= 143  49 (34.3%) | 5.24x10^-1^ |
| rs1342326  (*IL33*) | N= 472  165 (35%) | N= 229  93 (40.6%) | N= 30  17 (56.7%) | **3.1**x10^-2^ |
| rs2244012  (*RAD50*) | N= 419  150 (35.8%) | N= 256  100 (39.1%) | N= 44  20 (45.5%) | 3.74x10^-1^ |
| rs1773542  (*CD247*) | N= 99  32 (32.3%) | N= 321  125 (38.9%) | N= 310  117 (37.7%) | 4.91x10^-1^ |
| rs61816764  (FLG) | N= 642  248 (38.6%) | N= 88  27 (30.7%) | | 1.49x10^-1^ |
| rs1420101  (*IL1RL1*) | N= 238  91 (38.2%) | N= 349  126 (36.1%) | N= 143  58 (40.6%) | 6.36x10^-1^ |
| rs1106639  (*D2HGDH*) | N= 37  8 (21.6%) | N= 251  92 (36.7%) | N= 443  175 (39.5%) | 9.0x10^-2^ |
| rs532965  (*HLA-DQA1*) | N= 426  150 (35.2%) | N= 273  111 (40.7%) | N= 31  14 (45.2%) | 2.37x10^-1^ |
| rs9272629  (*HLA-DQB1*) | N= 83  35 (42.2%) | N= 247  85 (34.4%) | N= 265  265 (42.6%) | 2.25x10^-1^ |
| rs1847472  (*BACH2*) | N= 68  26 (38.2%) | N= 333  117 (35.1%) | N= 328  130 (39.6%) | 1.35x10^-1^ |
| rs13275219  (*MIR5708*) | N= 287  106 (36.9%) | N= 340  123 (36.2%) | N= 101  44 (43.6%) | 3.91x10^-1^ |
| rs10905279  (*GATA3*) | N= 262  100 (38.2%) | N= 350  128 (36.6%) | N= 119  47 (39.5%) | 8.29x10^-1^ |
| rs1444782  (*LOC101928272*) | N= 121  49 (40.5%) | N= 346  132 (38.2%) | N= 261  94 (36%) | 6.89x10^-1^ |
| rs28592983  (*MUC5AC*) | N= 304  113 (37.2%) | N= 329  126 (38.3%) | N= 98  36 (36.7%) | 9.40x10^-1^ |
| rs61893460  (*C11orf30*) | N= 218  72 (33%) | N= 333  131 (39.3%) | N= 179  72 (40.2%) | 2.35x10^-1^ |
| rs10876864  (*RPS26*) | N= 227  91 (40.1%) | N= 367  128 (34.9%) | N= 135  56 (41.5%) | 2.70x10^-1^ |
| rs10519067  (*RORA*) | N= 153  60 (39.2%) | | N= 578  215 (37.2%) | 6.47x10^-1^ |
| rs36045143  (*CLEC16A*) | N= 38  7 (18.4%) | N= 239  88 (36.8%) | N= 454  180 (39.6%) | **3.3**x10^-2^ |
| rs16948048  (*ZNF652*) | N= 257  94 (36.6%) | N= 367  129 (35.1%) | N= 104  49 (47.1%) | 8.0x10^-2^ |
| rs3806933  (*WDR36*) | N= 131  51 (38.9%) | N= 329  125 (38%) | N= 270  99 (36.7%) | 8.96x10^-1^ |
| rs1837253  (*TSLP*) | N= 29  13 (44.8%) | N= 254  87 (34.3%) | N= 446  173 (38.8%) | 3.46x10^-1^ |
| rs17293632  (*SMAD3*) | N= 374  153 (40.9%) | N= 314  104 (33.1%) | N= 42  18 (42.9%) | 8.5x10^-2^ |
| rs17388568  (*KIAA1109*) | N= 397  153 (38.5%) | N= 282  98 (34.8%) | N= 50  23 (46%) | 2.69x10^-1^ |
| rs9303277  (*IKZF3*) | N= 183  74 (40.4%) | N= 348  134 (38.5%) | N= 198  67 (33.8%) | 3.80x10^-1^ |

**Supplementary Table 11 Genetic signals identified in the moderate to severe asthma GWAS and their association with blood eosinophil count:** data is presented as percentage and n’s for the patients who had a blood eosinophil count of >300 µL and then the total number of patients who had a blood eosinophil count across the risk allele groups for moderate-severe asthma with 0 indicating carrying no risk alleles for moderate-severe asthma, 1 carrying 1 risk allele for moderate-severe asthma and 2 carrying 2 risk alleles for moderate-severe asthma. A chi-squared test was used to analyse both these categorical groups. Bonferroni correction p= <2x10^-3^.

| Locus | 0 | 1 | 2 | P-value | Power |
| --- | --- | --- | --- | --- | --- |
| rs10077785  *C5orf56* | N= 331  2.21 (0.81) | N= 241  2.19 (0.83) | N= 41  2.24 (0.91) | 9.29x10^-1^ | 0.35 |
| rs167769  *STAT6* | N= 190  2.27 (0.89) | N= 298  2.16 (0.80) | N= 124  2.19 (0.76) | 3.53x10^-1^ | 1.0 |
| rs1342326  *IL-33* | N= 395  2.19 (0.81) | N= 201  2.24 (0.85) | N= 15  1.97 (0.68) | 4.35x10^-1^ | 1.0 |
| rs2244012  *RAD50* | N= 375  2.22 (0.80) | N= 207  2.18 (0.85) | N= 30  2.19 (0.86) | 8.68x10^-1^ | 0.89 |
| rs1773542  *CD247* | N= 112  2.17 (0.79) | N= 275  2.17 (0.81) | N= 224  2.26 (0.84) | 4.29x10^-1^ | 1.0 |
| rs61816764  *FLG** | N= 533  2.18 (0.82) | N= 78  2.34 (0.81) | | 1.06x10^-1^ | 0.30 |
| rs1420101  *IL1RL1* | N= 112  2.18 (0.80) | N= 272  2.16 (0.81) | N= 229  2.25 (0.85) | 4.38x10^-1^ | 1.0 |
| rs1106639  *D2HGDH* | N= 27  2.03 (0.88) | N= 199  2.26 (0.84) | N= 384  2.18 (0.81) | 3.0x10^-1^ | 1.0 |
| rs532965  *HLA-DQA1* | N= 362  2.15 (0.82) | N= 212  2.26 (0.82) | N= 36  2.35 (0.85) | 1.43x10^-1^ | 1.0 |
| rs9272629  *HLA-DQB1* | N= 114  2.23 (0.82) | N= 186  2.18 (0.78) | N= 301  2.21 (0.85) | 8.3x10^-1^ | 0.99 |
| rs1847472  *BACH2* | N= 63  2.27 (0.85) | N= 274  2.21 (0.88) | N= 274  2.17 (0.75) | 6.77x10^-1^ | 1.0 |
| rs13275219  *MIR5708* | N= 241  2.21 (0.82) | N= 291  2.19 (0.83) | N= 78  2.20 (0.79) | 9.55x10^-1^ | 0.16 |
| rs10905279  *GATA3* | N= 223  2.23 (0.84) | N= 304  2.19 (0.74) | N= 85  2.15 (0.86) | 7.36x10^-1^ | 1.0 |
| rs1444782  *LOC101928272* | N= 70  2.23 (0.70) | N= 316  2.17 (0.85) | N= 224  2.22 (0.81) | 7.61x10^-1^ | 1.0 |
| rs28592983  *MUC5AC* | N= 255  2.23 (0.83) | N= 278  2.18 (0.87) | N= 75  2.18 (0.64) | 7.89x10^-1^ | 1.0 |
| rs61893460  *C11orf30* | N= 154  2.32 (0.77) | N= 297  2.19 (0.85) | N= 151  2.14 (0.81) | 1.46 x10^-1^ | 1.0 |
| rs10876864  *RPS26* | N= 174  2.13 (0.88) | N= 319  2.23 (0.80) | N= 117  2.23 (0.81) | 4.40x10^-1^ | 1.0 |
| rs10519067  *RORA* | N= 109  2.21 (0.88) | | N= 503  2.20 (0.81) | 9.08x10^-1^ | 0.05 |
| rs36045143  *CLEC16A* | N= 40  2.11 (0.75) | N= 213  2.14 (0.82) | N= 360  2.25 (0.83) | 2.30x10^-1^ | 1.0 |
| rs16948048  *ZNF652* | N= 217  2.25 (0.84) | N= 301  2.18 (0.85) | N= 91  2.13 (0.67) | 4.42x10^-1^ | 1.0 |
| rs3806933  *WDR36* | N= 89  2.25 (0.82) | N= 282  2.16 (0.85) | N= 242  2.22 (0.79) | 5.29x10^-1^ | 1.0 |
| rs1837253  *TSLP* | N= 31  2.37 (0.90) | N= 206  2.20 (0.76) | N= 376  2.18 (0.85) | 4.77x10^-1^ | 1.0 |
| rs17293632  *SMAD3* | N= 331  2.22 (0.84) | N= 239  2.18 (0.79) | N= 42  2.11 (0.92) | 6.31x10^-1^ | 1.0 |
| rs17388568  *KIAA1109* | N= 292  2.17 (0.81) | N= 272  2.20 (0.83) | N= 48  2.39 (0.86) | 2.17x10^-1^ | 1.0 |
| rs9303277  *IKZF3* | N= 143  2.14 (0.88) | N= 275  2.25 (0.78) | N= 165  2.20 (0.79) | 3.63x10^-1^ | 1.0 |

**Supplementary Table 12 Genetics signals identified in the moderate to severe asthma GWAS study did not drive the levels of IFN-ƴ in the serum of asthma patients:** data is presented as mean log_10_ pg/mL and standard deviation. ANOVA was used to analyse the 3 genotype groups. *Two genotyping groups were combined due to the n’s being low in one of the groups therefore independent T-tests was used analyses. Power was also computed and a value more than 0.8 is considered a well powered study. Bonferroni correction p= <2x10^-3^.

| Locus | 0 | 1 | 2 | P-value | Power |
| --- | --- | --- | --- | --- | --- |
| rs10077785  *C5orf56* | N= 324  1.54 (0.57) | N= 234  1.57 (0.59) | N= 38  1.58 (0.51) | 7.92x10^-1^ | 1.0 |
| rs167769  *STAT6* | N= 179  1.60 (0.60) | N= 298  1.54 (0.57) | N= 117  1.53 (0.55) | 5.08x10^-1^ | 1.0 |
| rs1342326  *IL-33* | N= 382  1.57 (0.60) | N= 195  1.55 (0.52) | N= 16  1.37 (0.54) | 3.93x10^-1^ | 1.0 |
| rs2244012  *RAD50* | N= 372  1.55 (0.58) | N= 192  1.56 (0.57) | N= 30  1.57 (0.50) | 9.74x10^-1^ | 0.08 |
| rs1773542  *CD247* | N= 108  1.54 (0.55) | N= 268  1.52 (0.60) | N= 216  1.60 (0.55) | 2.62x10^-1^ | 1.0 |
| rs61816764  *FLG** | N= 519  1.54 (0.58) | N= 73  1.68 (0.54) | | 4.4x10^-2^ | 0.54 |
| rs1420101  *IL1RL1* | N= 110  1.52 (0.56) | N= 265  1.61 (0.60) | N= 220  1.6 (0.55) | 2.59x10^-1^ | 1.0 |
| rs1106639  *D2HGDH* | N= 25  1.36 (0.54) | N= 192  1.56 (0.55) | N= 375  1.56 (0.59) | 2.24x10^-1^ | 1.0 |
| rs532965  *HLA-DQA1* | N= 354  1.56 (0.57) | N= 207  1.56 (0.58) | N= 31  1.45 (0.55) | 5.74x10^-1^ | 1.0 |
| rs9272629  *HLA-DQB1* | N= 115  1.63 (0.58) | N= 180  1.57 (0.54) | N= 289  1.53 (0.59) | 2.41x10^-1^ | 1.0 |
| rs1847472  *BACH2* | N= 58  1.65 (0.60) | N= 271  1.56 (0.57) | N= 264  1.53 (0.57) | 3.26x10^-1^ | 1.0 |
| rs13275219  *MIR5708* | N= 229  1.55 (0.55) | N= 285  1.56 (0.59) | N= 79  1.56 (0.59) | 9.84x10^-1^ | 0.06 |
| rs10905279  *GATA3* | N= 220  1.58 (0.58) | N= 290  1.54 (0.57) | N= 84  1.53 (0.58) | 6.51x10^-1^ | 1.0 |
| rs1444782  *LOC101928272* | N= 75  1.62 (0.60) | N= 299  1.57 (0.56) | N= 218  1.57 (0.56) | 3.92x10^-1^ | 1.0 |
| rs28592983  *MUC5AC* | N= 254  1.56 (0.59) | N= 265  1.54 (0.56) | N= 72  1.57 (0.53) | 9.26x10^-1^ | 0.37 |
| rs61893460  *C11orf30* | N= 158  1.61 (0.54) | N= 273  1.53 (0.63) | N= 154  1.55 (0.51) | 3.69x10^-1^ | 1.0 |
| rs10876864  *RPS26* | N= 174  1.56 (0.62) | N= 305  1.56 (0.56) | N= 113  1.55 (0.55) | 9.97x10^-1^ | 0.05 |
| rs10519067  *RORA* | N= 107  1.65 (0.64) | | N= 487  1.54 (0.56) | 6.7x10^-2^ | 0.42 |
| rs36045143  *CLEC16A* | N= 35  1.49 (0.65) | N= 209  1.55 (0.57) | N= 351  1.57 (0.57) | 7.43x10^-1^ | 1.0 |
| rs16948048  *ZNF652* | N= 216  1.61 (0.57) | N= 284  1.53 (0.58) | N= 93  1.49 (0.55) | 1.32x10^-1^ | 1.0 |
| rs3806933  *WDR36* | N= 92  1.58 (0.49) | N= 276  1.53 (0.60) | N= 227  1.58 (0.57) | 5.95x10^-1^ | 1.0 |
| rs1837253  *TSLP* | N= 32  1.57 (0.44) | N= 198  1.55 (0.52) | N= 365  1.56 (0.61) | 9.50x10^-1^ | 0.19 |
| rs17293632  *SMAD3* | N= 309  1.55 (0.59) | N= 242  1.57 (0.57) | N= 43  1.56 (0.48) | 9.54x10^-1^ | 0.16 |
| rs17388568  *KIAA1109* | N= 280  1.53 (0.53) | N= 269  1.57 (0.61) | N= 46  1.62 (0.61) | 5.66x10^-1^ | 1.0 |
| rs9303277  *IKZF3* | N= 134  1.58 (0.65) | N= 273  1.54 (0.55) | N= 164  1.57 (0.56) | 8.29x10^-1^ | 0.98 |

**Supplementary Table 13 Genetics signals identified in the moderate to severe asthma GWAS study did not drive the levels of IL-4 in the serum of asthma patients:** data is presented as mean log_10_ pg/mL and standard deviation. ANOVA was used to analyse the 3 genotype groups. *Two genotyping groups were combined due to the n’s being low in one of the groups therefore independent T-tests was used analyses. Power was also computed and a value more than 0.8 is considered a well powered study. Bonferroni correction p= <2x10^-3^.

| Locus | 0 | 1 | 2 | P-value | Power |
| --- | --- | --- | --- | --- | --- |
| rs10077785  *C5orf56* | N= 216  0.66 (0.74) | N= 160  0.65 (0.83) | N= 29  0.66 (0.77) | 9.96x10^-1^ | 0.05 |
| rs167769  *STAT6* | N= 129  0.71 (0.81) | N= 200  0.64 (0.75) | N= 77  0.58 (0.80) | 4.62x10^-1^ | 1.0 |
| rs1342326  *IL-33* | N= 262  0.69 (0.81) | N= 134  0.59 (0.71) | N= 9  0.32 (0.91) | 2.21x10^-1^ | 1.0 |
| rs2244012  *RAD50* | N= 248  0.67 (0.78) | N= 137  0.64 (0.80) | N= 21  0.53 (0.67) | 7.01x10^-1^ | 1.0 |
| rs1773542  *CD247* | N= 66  0.68 (0.83) | N= 178  0.62 (0.81) | N= 159  0.68 (0.74) | 7.62x10^-1^ | 1.0 |
| rs61816764  *FLG** | N= 350  0.64 (0.79) | N= 55  0.74 (0.72) | | 3.98x10^-1^ | 0.14 |
| rs1420101  *IL1RL1* | N= 67  0.70 (0.82) | N= 177  0.62 (0.81) | N= 163  0.67 (0.74) | 7.36x10^-1^ | 1.0 |
| rs1106639  *D2HGDH* | N= 16  0.68 (0.83) | N= 135  0.68 (0.77) | N= 252  0.65 (0.78) | 9.34x10^-1^ | 0.21 |
| rs532965  *HLA-DQA1* | N= 248  0.63 (0.79) | N= 133  0.71 (0.74) | N= 22  0.60 (0.94) | 6.08x10^-1^ | 1.0 |
| rs9272629  *HLA-DQB1* | N= 81  0.76 (0.74) | N= 131  0.63 (0.77) | N= 184  0.65 (0.80) | 4.61x10^-1^ | 1.0 |
| rs1847472  *BACH2* | N= 43  0.72 (0.77) | N= 175  0.73 (0.77) | N= 187  0.57 (0.79) | 1.6x10^-1^ | 1.0 |
| rs13275219  *MIR5708* | N= 157  0.66 (0.73) | N= 193  0.66 (0.81) | N= 56  0.58 (0.81) | 7.85x10^-1^ | 0.99 |
| rs10905279  *GATA3* | N= 145  0.71 (0.68) | N= 206  0.64 (0.81) | N= 54  0.54 (0.86) | 3.76x10^-1^ | 1.0 |
| rs1444782  *LOC101928272* | N= 52  0.73 (0.85) | N= 202  0.60 (0.79) | N= 149  0.71 (0.74) | 3.43x10^-1^ | 1.0 |
| rs28592983  *MUC5AC* | N= 174  0.67 (0.76) | N= 178  0.67 (0.80) | N= 51  0.57 (0.75) | 6.83x10^-1^ | 1.0 |
| rs61893460  *C11orf30* | N= 107  0.67 (0.75) | N= 189  0.61 (0.85) | N= 104  0.72 (0.68) | 5.34x10^-1^ | 1.0 |
| rs10876864  *RPS26* | N= 119  0.63 (0.82) | N= 213  0.65 (0.75) | N= 74  0.71 (0.81) | 8.01x10^-1^ | 0.98 |
| rs10519067  *RORA* | N= 84  0.74 (0.86) | | N= 321  0.63 (0.76) | 3.05x10^-1^ | 0.17 |
| rs36045143  *CLEC16A* | N= 23  0.77 (0.91) | N= 141  0.66 (0.72) | N= 242  0.64 (0.80) | 7.58x10^-1^ | 1.0 |
| rs16948048  *ZNF652* | N= 146  0.68 (0.78) | N= 205  0.66 (0.77) | N= 53  0.58 (0.80) | 7.1x10^-1^ | 1.0 |
| rs3806933  *WDR36* | N= 60  0.79 (0.77) | N= 179  0.66 (0.76) | N= 167  0.60 (0.81) | 2.7x10^-1^ | 1.0 |
| rs1837253  *TSLP* | N= 24  0.65 (0.70) | N= 130  0.68 (0.75) | N= 253  0.64 (0.80) | 9.12x10^-1^ | 0.36 |
| rs17293632  *SMAD3* | N= 220  0.63 (0.80) | N= 159  0.71 (0.78) | N= 26  0.57 (0.57) | 5.17x10^-1^ | 1.0 |
| rs17388568  *KIAA1109* | N= 196  0.63 (0.76) | N= 173  0.71 (0.79) | N= 35  0.57 (0.83) | 4.5x10^-1^ | 1.0 |
| rs9303277  *IKZF3* | N= 95  0.70 (0.94) | N= 179  0.67 (0.71) | N= 106  0.66 (0.77) | 9.4x10^-1^ | 0.17 |

**Supplementary Table 14 Genetics signals identified in the moderate to severe asthma GWAS study did not drive the levels of IL17A in the serum of asthma patients:** data is presented as mean log_10_ pg/mL and standard deviation. ANOVA was used to analyse the 3 genotype groups. *Two genotyping groups were combined due to the n’s being low in one of the groups therefore independent T-tests was used analyses. Power was also computed and a value more than 0.8 is considered a well powered study. Bonferroni correction p= <2x10^-3^.
